# An AI‐assisted integrated, scalable, single‐cell phenomic‐transcriptomic platform to elucidate intratumor heterogeneity against immune response

**DOI:** 10.1002/btm2.10628

**Published:** 2024-01-02

**Authors:** Christopher P. Tostado, Lucas Xian Da Ong, Joel Jia Wei Heng, Carlo Miccolis, Shumei Chia, Justine Jia Wen Seow, Yi‐Chin Toh, Ramanuj DasGupta

**Affiliations:** ^1^ Genome Institute of Singapore, Laboratory of Precision Oncology and Cancer Evolution Singapore Singapore; ^2^ Institute for Health Innovation and Technology (iHealthtech), National University of Singapore Singapore Singapore; ^3^ School of Mechanical, Medical and Process Engineering Queensland University of Technology Brisbane Australia; ^4^ Centre for Biomedical Technologies Queensland University of Technology Brisbane Australia

**Keywords:** computer vision, head and neck cancer, immunotherapy, microfluidics, precision oncology, single‐cell transcriptomics

## Abstract

We present a novel framework combining single‐cell phenotypic data with single‐cell transcriptomic analysis to identify factors underpinning heterogeneity in antitumor immune response. We developed a pairwise, tumor‐immune discretized interaction assay between natural killer (NK‐92MI) cells and patient‐derived head and neck squamous cell carcinoma (HNSCC) cell lines on a microfluidic cell‐trapping platform. Furthermore we generated a deep‐learning computer vision algorithm that is capable of automating the acquisition and analysis of a large, live‐cell imaging data set (>1 million) of paired tumor‐immune interactions spanning a time course of 24 h across multiple HNSCC lines (*n* = 10). Finally, we combined the response data measured by Kaplan–Meier survival analysis against NK‐mediated killing with downstream single‐cell transcriptomic analysis to interrogate molecular signatures associated with NK‐effector response. As proof‐of‐concept for the proposed framework, we efficiently identified MHC class I‐driven cytotoxic resistance as a key mechanism for immune evasion in nonresponders, while enhanced expression of cell adhesion molecules was found to be correlated with sensitivity against NK‐mediated cytotoxicity. We conclude that this integrated, data‐driven phenotypic approach holds tremendous promise in advancing the rapid identification of new mechanisms and therapeutic targets related to immune evasion and response.

**Translational Impact Statement**

Our unique and automation‐amenable data‐driven phenotyping approach, combining a microfluidic cell‐pairing assay, AI‐driven computer vision, and downstream single‐cell transcriptomic analysis to uncover head and neck cancer line heterogeneity against natural killer cell cytotoxicity provides a strong alternative method for detection of patient‐specific heterogeneity and can potentially act as a tool for accelerating biomarker discovery and combinatorial immunotherapy testing.

## 1 INTRODUCTION

Recent advances in cancer immunotherapy have provided new avenues for treating a variety of different cancer types.^1^ The last decade has seen numerous FDA‐approved immunotherapy treatments used alone or in combination with other standard‐of‐care treatment to effectively combat various hematological cancers. There is now a strong desire to extend the success of these novel treatments to target solid tumors—such as squamous cell carcinomas of the head and neck and hepatocellular carcinomas—as seen by a growing number of ongoing clinical trials.^2^, ^3^, ^4^

These novel immune‐combinatorial treatments, which rely on the body's own immune response, often entail an added level of complexity. They require an understanding of not only the molecular signature of patients' tumors, but also the phenotypic response characterized by the interactions between the patients' tumor and immune cells and the tumor microenvironment (TME). Recently, new combinatorial immunotherapies have been applied for treating recurrent or metastatic squamous cell carcinoma (HNSCC), the most common type of head and neck cancer accounting for approximately 90% of diagnosed cases and with reported five‐year survivability of ~50–66%.^2^, ^5^ This low survivability is despite the development of new therapies; anti programmed death 1 (PD‐1) has shown some initial promising results and has been applied both for recurrent/metastatic as well as preoperative neoadjuvant treatment of untreated cases. However, effective patient selection remains a major challenge.^6^, ^7^, ^8^, ^9^ Stratification of predicted patient response is often based exclusively on a limited set of biomarkers such as PD‐ligand 1 (PD‐L1) or determined via extensive pan‐omic screening strategies applied in the context of clinical trials that are both time and cost‐intensive. Additionally, limited access to sufficient quantity of heterogeneous patient samples further impedes this process, especially in the early stages of biomarker discovery. There is therefore a clear, unmet need to expedite and scale testing and screening methods, especially in the face of a growing number of promising combinatorial therapeutic options.

In vitro patient‐derived models (primary cancer lines or tumor organoids) have been used for testing the transcriptomic and phenotypic response of tumors to a variety of different anticancer therapies,^10^, ^11^ and can be deployed in new ways as useful tools for rapid investigation of intra‐tumor or inter‐patient heterogeneity. More recently, such in vitro tumor cell/spheroid models have been utilized in microfluidic coculture settings, where they can be combined with immune cells to allow for a more controlled environment that provides a methodical approach to characterize and quantify interactions between target and effector cells.^12^, ^13^, ^14^ However, existing microfluidic coculture devices are designed to track and measure tumor‐immune interactions *en masse*. This generates minimal phenotypic data sets,^15^, ^16^ which are not amenable to statistical visualization and analysis techniques often employed in single cell transcriptomics to measure inter‐ or intra‐tumor heterogeneity.^17^, ^18^ Hence, it has not been possible to correlate phenotypic observations on immune‐mediated tumor killing with transcriptomics data to uncover potential molecular mediators. We postulate that this limitation can be circumvented by using microfluidic coculture devices that can partition tumor and immune cells into massively discretized interactions as exemplified by microfluidic hydrodynamic cell trapping devices. These devices are comprised of an ensemble of hundreds of individual cell traps aligned on a grid, with each trap capable of trapping live cells in confined spaces to track and measure individual, pairwise cellular interactions.^19^, ^20^, ^21^

**FIGURE 1:**
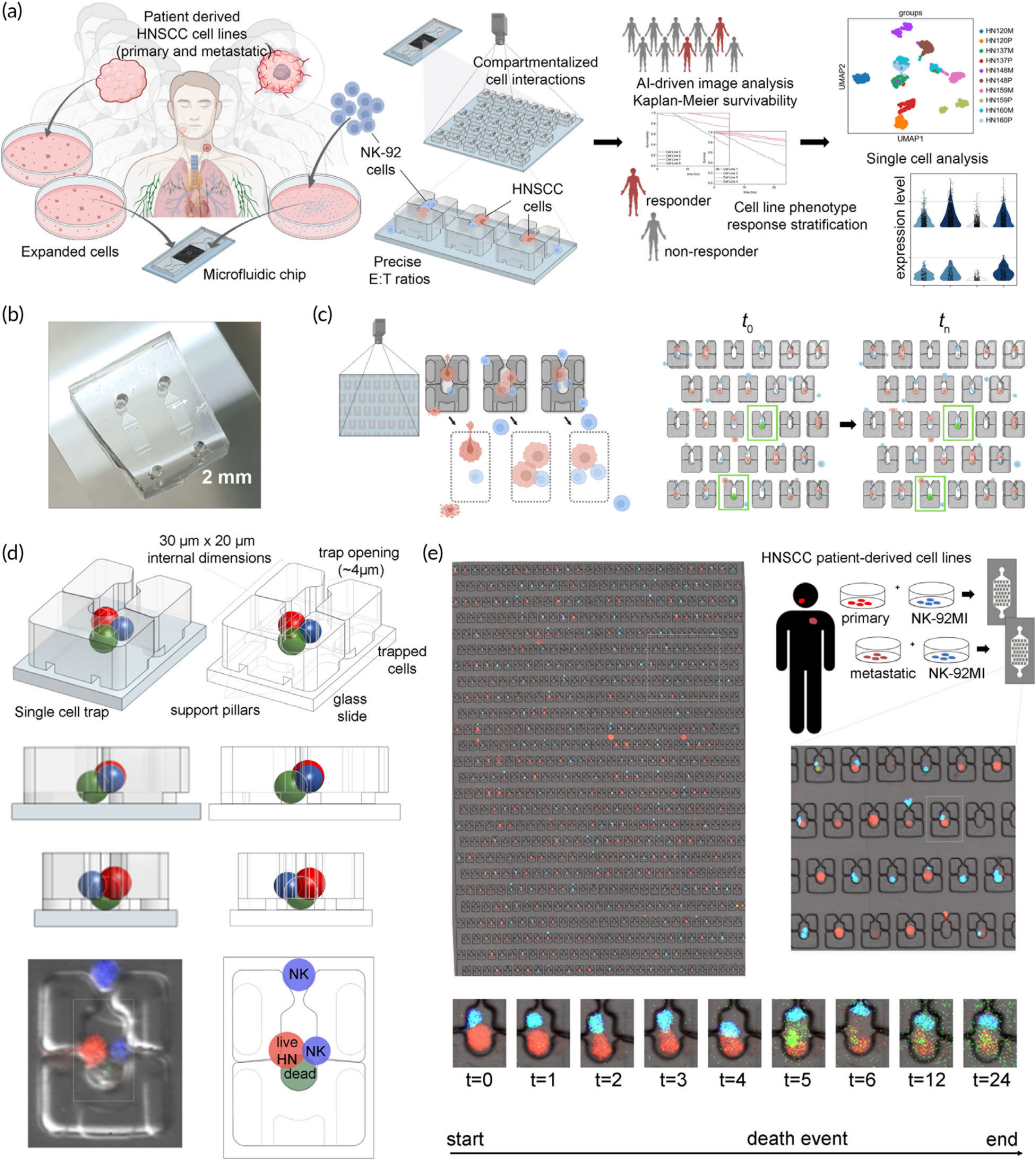
A framework for measuring head and neck squamous cell carcinoma (HNSCC) heterogeneity against natural killer (NK)‐mediated response. (a) Patient‐derived HNSCC cell line phenotypic response stratification was achieved using tumor‐immune discrete interaction (TIDI) experiments conducted in a microfluidic hydrodynamic cell trapping device and combined with downstream analysis. Ten different patient‐derived HNSCC cell lines were expanded, singularized and paired with NK‐92MI cells in hydrodynamic cell trap arrays on separate microfluidic chips at effector tumor (E:T) ratios ranging from 1:1 to 1:3. The 0:1 and 1:0 E:T ratios were used as on‐chip controls. The chip was comprised of an ensemble of traps in which precise E:T ratios could be monitored, tracked, and recorded. On‐chip response of each of the cell lines to NK‐mediated killing over a 24‐h period was determined using Kaplan Meier survivability analysis and HNSCC cell lines were designated as responders or nonresponders based on 24‐h survivability. Downstream single‐cell analysis was used to identify key differences in expression profiles to explain differences in response. (b) A photograph of the PDMS microfluidic device (scale bar = 2 mm). (c) Compartmentalization of the cells into separate cell traps allowed for precise region‐of‐interest (ROI) determination and tracking of cell death events over time. (d) A detailed schematic of the cell trapping structures. (e) A stitched micrograph of the ensemble of cell traps in a microfluidic chip. Precise ROIs were identified using a deep‐learning (DL) computer vision algorithm and these ROIs were used to crop the larger image into smaller images of individual traps which could be tracked over time. Cell death events were automatically recorded using a separate DL algorithm.

Here, we present a novel framework for measuring heterogeneity of patient‐derived cell lines in their response against innate, immune‐effector activity. We also highlight the potential of this platform to accelerate biomarker discovery by correlating single‐cell phenotypic effector‐tumor response data with single‐cell transcriptomics (Figure 1a). Using an existing multiplexed microfluidic cell trapping device to conduct single‐cell tumor‐immune discretized interaction (TIDI) experiments on‐chip, we pair 10 of our previously isolated and expanded patient‐derived HNSCC cell lines together with commercially available NK‐92 cells inside cell traps in defined E:T ratios of 1:1, 2:1, or 3:1. We demonstrate successful generation of phenotypic response profiles for these cell lines in the form of Kaplan–Meier survivability curves by using a modified pretrained residual network‐based deep learning (DL) algorithm to rapidly analyze tens of thousands of cellular interactions and track immune‐mediated killing events over a 24‐h period. We then compare the phenotypic response profiles to single‐cell transcriptomic analysis of the 10 cell lines to identify differences in their expression profiles between “responders” and “nonresponders.” We demonstrate that our unique streamlined workflow of integrating microfluidic device design for systematic interrogation of functional immune‐tumor interactions with AI‐driven image analysis and single cell gene expression studies can be used for rapid response‐based stratification of patient‐derived cancer cell lines, as well as for the cost‐efficient discovery of prognostic biomarkers, based on immune function‐modulating cell states.

## 2 RESULTS

### 2.1 HNSCC and NK‐92MI hydrodynamic cell trapping in a microfluidic device

Current microfluidic tumor‐immune coculture devices often control the compartmentalization of tumor and effector cell populations to mimic their interactions within the TME.^12^, ^13^, ^15^, ^22^, ^23^, ^24^ However, their designs are not catered for making phenotypic measurements of tumor‐immune cell interactions in a highly parallelized manner, which limits the data points that can be generated from each patient‐specific tumor or immune cell population. Here, we employed a previously developed single cell pairing device (Figure 1b), which embodies a class of microfluidic devices known as hydrodynamic cell trapping devices that are developed for applications, such as stem cell fusion, individual cell activation, and protein analysis.^25^ Each device contains an array of physical traps that are designed to immobilize two or more cells in close physical proximity to track their subsequent interactions with each other. Hence, tumor‐immune cell interactions in these microfluidic cell trapping devices occur as a large number of discretized cell pairs (>900 traps per device) instead of two interacting populations observed in existing tumor‐immune coculture devices. The effector‐to‐target (E:T) ratio for each trap can be tightly controlled by varying the dimensions of the cell traps, the seeding densities of tumor and immune cells, and the order in which they are seeded into the device.^20^ Cytotoxic tumor killing events within each cell trap can be individually tracked using live‐fluorescent probes for live cells and Caspase 3/7 activity and monitored longitudinally for up to 24 h (Figure 1c,d). Thus, with the cell trapping devices, we could significantly increase the phenotypic data points indicative of cytotoxic tumor killing measured per device (i.e., 600–900/device). Furthermore, we were able to minimize problems related with reproducibility and accuracy, typically associated with bulk immune‐tumor cocultures (Supplementary Figure S1a), that may arise due to localized, variable E:T ratios, difficulty in tracking specific cell interactions over time in a dynamic environment, and arbitrary user‐specific defined regions‐of‐interest (ROIs) that are often required for downstream image analysis.

Our aim was to utilize the microfluidic cell trapping device to generate a sufficiently large data set measuring immune cell mediated tumor killing events so that we can fit them into statistical models to quantitatively identify differences in the phenotypic response of a small cohort of patient‐derived cell lines to immune‐mediated killing. Ten different HNSCC cell lines were derived using an established pipeline to generate patient‐derived primary cell (PDCs) lines, reported previously in Reference 10. Both the PDC tumor lines and effector immune cells (NK‐92) were expanded in conventional tissue culture flasks, harvested, and prepared into single cell suspension for seeding into the microfluidic cell trapping devices. Prior to seeding, the HNSCC tumor cell lines and NK cells were fluorescently labeled with Cell Tracker Orange and Cell Tracker Violet, respectively to visualize the two cell types within the device. The HNSCC tumor cells were first loaded into the cell traps, followed by a washing step to remove excess tumor cells before NK cells were perfused into the device. Although the cell trapping process was stochastic,^20^ we could achieve 600–900 individual hydrodynamic trapping structures with discrete tumor‐immune cell pairing in a single device by optimizing the cell density and seeding flow rates. The dimensions of the cell trapping structures, and chip architecture are shown in Figure 1d,e while detailed information on the seeding process can be found in Supplementary Figure S2. Live‐cell imaging with confocal microscopy was used to monitor tumor‐NK interactions every 20 min for 24 h and a caspase‐3/7 green apoptosis detection reagent was used to track immune mediated killing events. The entire seeded microfluidic device as well as examples of NK‐mediated cytotoxicity observed during the live‐cell imaging experiments are shown in Figure 1e and in Supplementary Movies [Link], [Link]. We verified that the microfluidic cell trapping devices could maintain high end‐point cell viability for the HNSCC tumor cell lines for 24 h (Supplementary Figure S1b,c). On‐chip control populations for both cell types also demonstrated high survivability (Table 1 columns 12–13) showing no adverse effects on cells as results of on‐chip culture.

**TABLE 1:** Overview of microfluidic cell‐trapping experiments performed.

Experimental parameters			R‐CNN1 Output	R‐CNN2 output							OriginPro output				
Patient‐derived cell line	Run	Device architecture Rows × columns (total # of traps)	Image Set (No. unbroken traps × No. time points)	Number of occupied traps at each E:T Ratio							Survivability after 24‐h				
				Used for analysis					Not used						
				Single immune (control)	Single tumor (control)	1:1 ratio	2:1 ratio	3:1 ratio	Other ratios/ empty	Broken/ distorted	Single immune (control)	Single tumor (control)	1:1 ratio	2:1 ratio	3:1 ratio
HN 1 (137 M)	1	13 × 29 + 13 × 30 (767)	53,947 (739 × 73)	6	40	243	66	7	377	28	0.9167	1.00	0.9732	0.9512	1.00
	2	13 × 29 + 13 × 30 (767)	48,837 (669 × 73)	6	13	233	139	45	233	98					
HN 2 (120 M)	1	15 × 30 + 15 × 31 (915)	55,699 (763 × 73)	9	33	400	105	32	184	152	1.00	0.8421	0.8766	0.8407	0.9063
	2	30 × 29 (870)	60,006 (822 × 73)	32	24	386	165	32	183	48					
HN 3 (159 M)	1	30 × 29 (870)	56,420 (806 × 70)	9	21	426	127	17	206	64	1.00	0.9775	0.9064	0.8535	0.9231
	2	15 × 30 + 15 × 31 (915)	59,933 (821 × 73)	13	68	279	112	9	340	94					
HN 4 (159P)	1	30 × 29 (870)	61,904 (848 × 73)	8	13	177	129	45	441	22	1.00	1.00	0.8588	0.9070	0.8667
	2	30 × 29 (870)	47,523 (651 × 73)	69	91	213	79	12	187	219					
HN 5 (120P)	1	26 × 28 (728)	48,837 (669 × 73)	7	7	138	179	39	299	59	0.9286	0.8889	0.3439	0.3308	0.3693
	2	15 × 30 + 15 × 31 (915)	59,714 (818 × 73)	7	11	83	87	26	604	97					
HN 6 (160P)	1	26 × 28 (728)	52,925 (725 × 73)	28	187	271	82	7	150	3	0.9367	0.9223	0.8247	0.7154	0.6316
	2	26 × 28 (728)	36,719 (503 × 73)	51	79	117	48	12	196	225					
HN 7 (160 M)	1	13 × 29 + 13 × 30 (767)	53,801 (737 × 73)	26	109	277	108	18	199	30	0.9666	0.8877	0.8426	0.8572	0.8070
	2	13 × 29 + 13 × 30 (767)	53,290 (730 × 73)	4	87	244	109	39	247	37					
HN 8 (148P)	1	15 × 30 + 15 × 31 (915)	63,364 (868 × 73)	6	11	286	146	44	375	47	1.00	0.8571	0.8534	0.855	8077
	2	13 × 29 + 13 × 30 (767)	54,385 (745 × 73)	29	143	294	54	8	217	22					
HN 9 (148 M)	1	30 × 29 (870)	53,509 (733 × 73)	18	248	263	24	1	179	137	1.00	0.9960	0.9924	1.00	1.00
HN 10 (137P)	1	15 × 30 + 15 × 31 (915)	61,101 (837 × 73)	15	5	274	188	39	316	78	1.00	1.00	0.7409	0.7103	0.6441
	2	15 × 30 + 15 × 31 (915)	51,246 (702 × 73)	15	13	251	102	20	301	213					

### 2.2 Dynamic cell type and status tracking using DL

The use of the microfluidic trapping device for conducting tumor‐immune interaction assays potentially addresses the drawbacks of using population‐based tumor‐immune coculture models, but it also introduces an enormous amount of imaging data. A comparison of all 10 HNSCC cell lines with 24‐h live‐cell imaging experiments (*n* = 2) at 20‐min intervals generated more than 1 million time‐sequenced trap images. Manual analysis of these images would be unfeasible and defeat the purpose of a fast, microfluidic approach to stratification of patient cell‐line phenotypes. However, the design of the microfluidic cell trapping device is extremely amenable to automated image analysis with computer vision due to the fixed positioning of the cell traps which define the ROIs in which cellular interactions are potentially taking place.

**FIGURE 2:**
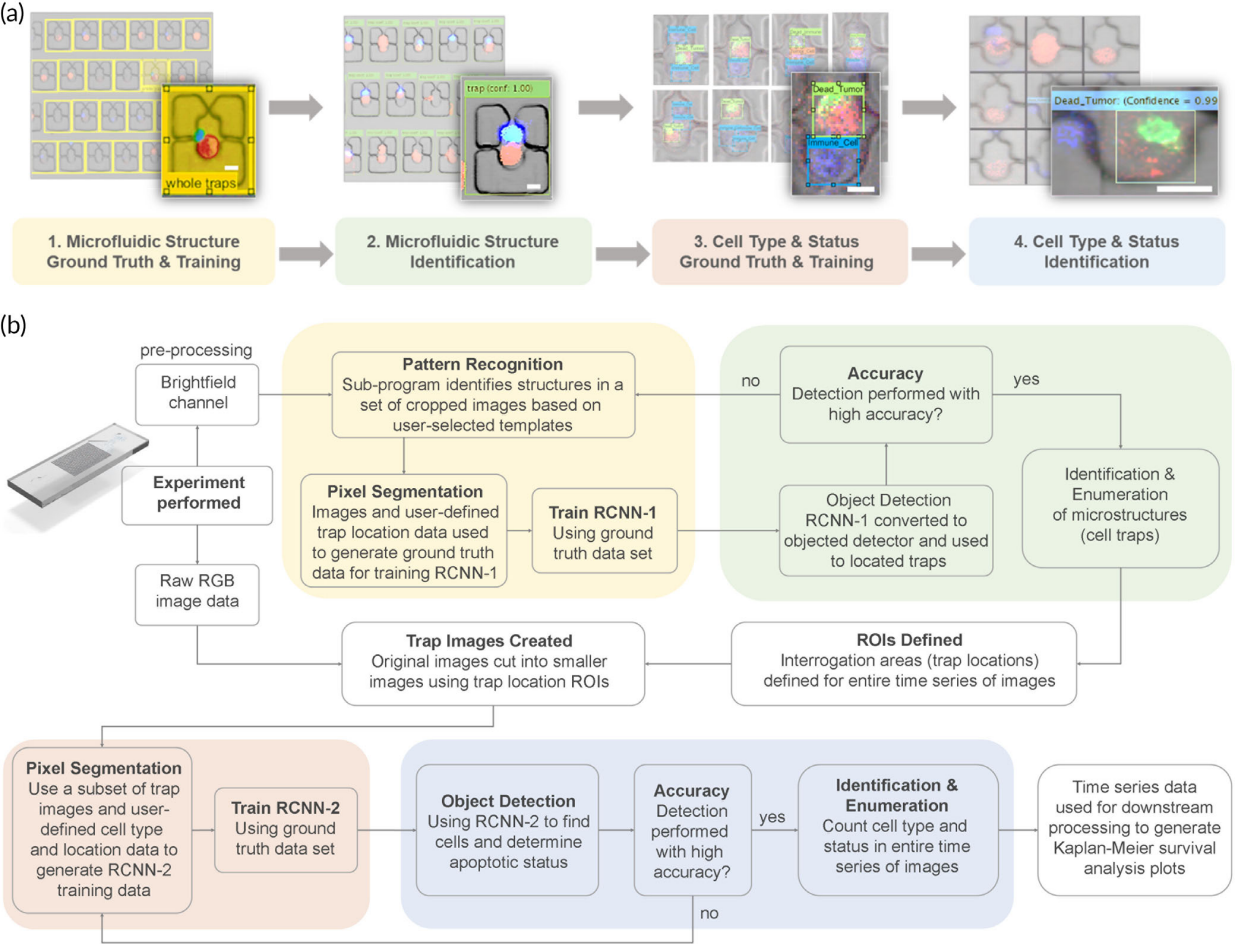
Dual deep learning computer vision algorithms deployed for automated trap identification and cytotoxicity analysis. (a) Automated computer vision analysis was divided into four stages: (1) the generation of a microfluidic structure ground truth data set and training of the first regions with convolutional neural network (RCNN‐1) to identify microfluidic cell trapping structures, (2) the deployment of RCNN‐1 to identify and enumerate relevant cell traps in the actual experimental data set, (3) the generation of a cell type (i.e., tumor cell, NK cell) and status (i.e., dead, live) ground truth data set and training of RCNN‐2 to identify cell death events indicated with caspase 3/7 reporter dye, and (4) deployment of RCNN‐2 to track and quantify cell death events in experimental data sets. (b) A flowchart detailing the specific steps taken at each stage in the computer vision analysis process.

Deep learning convolutional neural networks (CNNs) have been widely used to tackle classification problems in recent years. CNNs incorporating residual layers have been recognized as being particularly suited for efficient image classification and object tracking.^26^ Well established DL network architectures have been applied to image analysis, even in the immunotherapy and tumor‐immune interaction domains, although the applications have largely been restricted to histopathology or immunohistochemistry analysis and rarely include the systematic use of microfluidic platforms for precision control of cellular interactions.^27^, ^28^ Here, we integrated an automated computer vision approach for the analysis of cellular interactions occurring in a large array of individual cell traps found within the microfluidic trapping device by incorporating two separate DL residual CNNs. These neural networks were pretrained Resnet‐50 networks, a type of CNN incorporating residual layers, which we modified to incorporate region proposals (for object detection), thus creating region‐based CNNs (R‐CNNs). The first neural network was used to learn and identify the cell trapping microstructures used in the microfluidic device while the second was used to identify and enumerate cell type and status for the cells in each of the individual traps. All algorithms were programmed in‐house using MATLAB R2021a. A three‐dimensional diagram showing the basic architecture (feature maps and dimension) of the residual CNN (Resnet‐50) used for this study—consisting of a 224 × 224 × 4 pixel image input layer and five convolution blocks followed by an average pooling layer, a fully connected layer and a softmax layer for classification as well as the detailed network architecture and modifications made to the networks can be found in Supplementary Figure S3. The automated analysis procedure comprised four main steps: (1) microfluidic structure ground truth generation and training of the first network (RCNN‐1), (2) microfluidic structure identification, (3) cell type and status ground truth generation and training of the second network (RCNN‐2), and (4) cell type and status identification (Figure 2a). A basic workflow for the automated analysis procedure is shown in Figure 2b. For each 24‐h live‐cell imaging experiment, the entire microfluidic device was imaged by stitching together 20 (5 × 4) tiles of images to yield 72 stitched images (one image taken at time *t* = 0 and every subsequent 20‐min interval for 24 h) with each image approximately 3796 × 4641 pixels in size. Confocal imaging was conducted using four different channels: one for each of the labeled cell types (tumor and NK), one for the caspase 3/7 apoptosis green detection reagent, and one for the brightfield. For all experiments, raw bright‐field confocal image data were exported as a single channel and used separately to train the first network, RCNN‐1, for the identification of microfluidic structures. Preprocessing of the raw confocal image data obtained from the 24‐h live cell imaging experiments conducted on‐chip was required as a first step. From the bright‐field data set, >1000 image regions (691 × 751 pixels) were randomly selected and cropped from the larger images. These cropped images were provided to an in‐house written pattern‐recognition subprogram, which automatically generated the ground truth training data set with minimal user input. This training data set comprised of images matched with semantic image segmentation data describing the locations of traps in the images. The training set also underwent an augmentation step involving mirror image transformations and slight rotations to increase training set robustness. RCNN‐1 was a pretrained CNN from MATLAB with layer architecture based off Resnet‐50. The network was trained on more than a million images from the ImageNet (http://www.image-net.org) database to recognize and classify 1000 different common objects. Because the network has already learned to recognize many complex feature representations for a variety of images, it served as an excellent base network for transfer learning (i.e., using the features in images the network already recognizes such as lines, curves and shapes to identify new objects that were not part of its original training or identifiable classes). The transfer learning protocol (described in detail in Supplementary Figure S4) involved changing the number of classes which the network can identify, as well as replacing the last three layers of the network (the fully connected layer, the softmax layer, and the classification layer) with new untrained versions of the layers. Conversion of the network into an object detector also required several additional steps (Supplementary Figure S3c,d). After making the requisite modifications, RCNN‐1 was trained using the training data set according to the training conditions and computer hardware configurations described in Supplementary Table S1. The trained network successfully identified and tracked >95% of traps in a full test set comprised of a series of 73 (4343 × 3232 pixels) images. Detailed results for the training are found in Supplementary Figures S4 and S5 and Supplementary Movies [Link], [Link]

After completing steps 1 and 2 in the automated analysis workflow (Figure 2a), the position of each of the non‐broken, trackable cell traps in each image was determined, and the ROIs for the cellular interactions taking place on chip were fully defined. This ROI information was used to crop the raw stitched RGB confocal image data into a set of smaller images 56 × 72 pixels (W × H) in size. Each time‐stamped frame in the 24‐h experiments was automatically cut into smaller images and these images were then grouped according to trap number. The final output of RCNN‐1 for each experiment was a large data set of cropped, organized, sequential trap images defined as:

(1) $$\text{image dataset}=\sum_{t_{i}=1}^{i=1,2,3\ldots m}{\text{unbroken trap}}_{t}$$

Here, *m* is the total number of timepoints for the experiment. The output of RCNN‐1 is shown in Table 1 (column 4). A random subset of 3200 cropped trap images were used for training a separate network (RCNN‐2) which was used for identifying cell type and status of the cells in the traps. These images were labeled and saved as ground truth training images using the native MATLAB image labeler application. RCNN‐2 was created and modified in the same way as RCNN‐1 (Supplementary Figures S3 and S4). However, whereas RCNN‐1 was trained specifically to identify a single class (a microfluidic cell trap structure), RCNN‐2 was trained to recognize four separate classes: live/apoptotic HNSCC cells and live/apoptotic NK92‐MI cells (Figure 2a). RCNN‐2 analyzed the output of RCNN‐1 (the image set) to determine the number of occupied traps, the E:T ratio in each trap, and the occurrence of NK‐mediated apoptosis within the traps. The output of RCNN‐2 is shown in Table 1 (columns 5–10). Detailed results for training RCNN‐2 are found in Supplementary Table S1 and Supplementary Figure S6. Data output from RCNN‐2 was directly saved in spreadsheet format for easy downstream processing using Origin(Pro) version 2021b and can be found in supplementary data Table S3.

### 2.3 Immune mediated‐killing and survivability dependence on tumor‐immune ratio

**FIGURE 3:**
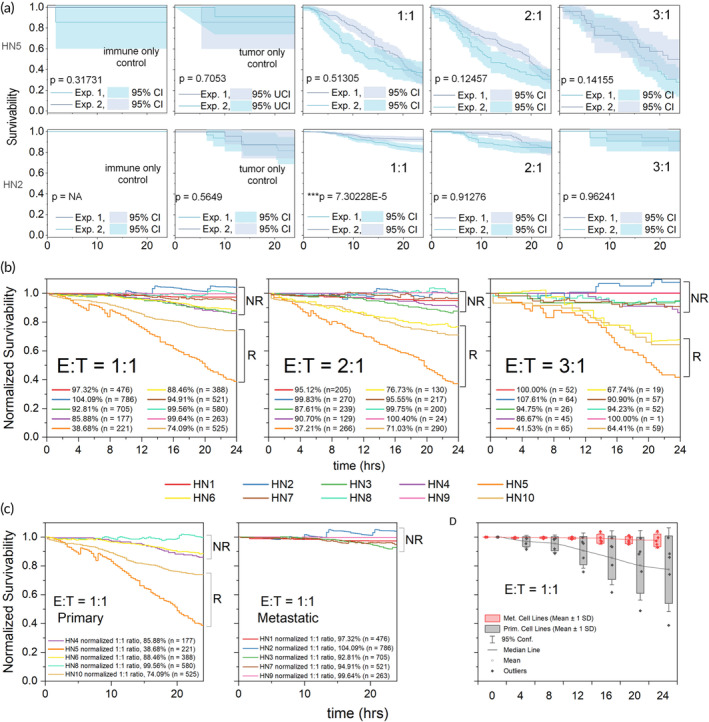
Phenotypic response data for 10 head and neck squamous cell carcinoma (HNSCC) cell lines using Kaplan–Meier survivability analysis. (a) On‐chip cell trapping experiments involved the identification and tracking of cell traps containing effector: tumor (E:T) ratios of interest, 1:1, 2:1, and 3:1, or relevant immune only (1:0) and tumor only (0:1) controls. Kaplan–Meier survivability was calculated for each group across a 24‐h on‐chip incubation period. Survivabilities and specific number of corresponding traps is shown. Duplicate cell trapping experiments conducted for two different representative HNSCC cell lines, HN2 and HN5, exhibited close replicability despite the use of slight differences in the microfluidic chips used to conduct experiments. (b) Normalized survivability using on‐chip control population survivability was calculated for all 10 HNSCC cell lines at different E:T ratios and cell lines were categorized as either nonresponders (NR) or responders (R). (c) HNSCC cell lines grouped according to tumor origin (primary or met) for E:T = 1:1. Not all primary cell lines were responders, but all responders were in the primary cell line group. All metastatic cell lines were nonresponders. (d) Survivability variance and the onset of variability among primary cell lines was greater and earlier (respectively) than that of the metastatic cell lines.

Having completed the training for both networks, using RCNN‐1 for determining dynamic trap structure identification and positioning (ROIs), and RCNN‐2 for cell type (blue‐stained NK92‐MI or red‐stained HNSCC) and status (live or apoptotic) determination, we could then deploy the networks to examine the relationship between immune cell mediated‐killing within a 24‐h period, on‐chip survivability, and E:T ratio. Each trap in the microfluidic chip corresponded to a single “micro” experiment representing one potential cellular interaction at a specific E:T ratio. These interactions were tracked, subdivided into populations based on E:T ratios of interest and analyzed using RCNN‐1 and RCNN‐2. By treating each immune mediated tumor death occurring in a cell trap as an individual “event,” we will have sufficient data points to fit into a Kaplan–Meier survival curve for each HNSCC line. This allowed us to statistically stratify the different phenotypic responses of the patient tumor cell lines to NK‐92MI exposure at different E:T ratios. Notably, this stratification could be used as a rapid and accessible reference for downstream transcriptomic profiling. The Kaplan–Meier survival probability step functions for the E:T = 1:0, 0:1, 1:1, 2:1, and 3:1 ratio populations on‐chip for each of the HNSCC cell lines were calculated according to:

(2) $$S\left(t\right)=\prod_{t_{i}=1}^{i=1,2,3..m}\left[\frac{n_{t}-d_{t}}{n_{t}}\right]$$

where *n*_*t*_ is the sample number for that population at time *t*_*i*_, *m* is the number of timepoints, and *d*_*t*_ is the number of observed events and censored observations at time *t*_*i*_. Using a log‐rank test statistic to compare survivability analysis results,^29^ we observed no (or very limited) significant difference between duplicate experiments (Figure 3a). Therefore, the corresponding E:T populations in each duplicate experiment were combined for the statistic fitting. To account for slight variations in viability and the relative risk of cell death occurring normally due to on‐chip cell culture, the survivability for on‐chip populations of 1:1, 2:1, and 3:1 (E:T) ratios were normalized relative to the corresponding 0:1 E:T on‐chip tumor‐only control population prior to comparison to determine differences in immune‐mediated killing across all 10 HNSCC tumor cell lines (Figure 3b). Considering that only slight differences were observed between 1:1, 2:1, and 3:1 ratio survivabilities, the survivabilities at the E:T = 1:1 ratio were assumed to be sufficient to provide a reasonable estimate of cell line response. Here, we designate cell lines exhibiting <75% survivability after 24 h as responders, while those cell lines with higher 24‐h survivabilities are classified as nonresponders HN5 and HN10 exhibited survivabilities of ~40 and ~75%, respectively, and were labeled as having a significant response to NK cytotoxic killing.

### 2.4 Primary versus metastatic HNSCC cell line response to NK cells

Having classified the response of individual cell lines to immune mediated killing based on E:T ratio, we then sought to determine whether there was a correlation between response and tissue origin of the individual HNSCC cell lines. For this study, the patient‐derived HNSCC lines were chosen such that there was an equal representation of primary and lymph‐node metastatic samples among cell lines. The cell lines were plotted according to cell line origin and compared to determine if origin could be used as a general prognosticator for survivability profile (Figure 3c). Cell lines which exhibited survivability <80% (HN5 and HN10) were deemed responders to NK‐mediated cytotoxicity, while all other cell lines were deemed as resistant and categorized as nonresponders. The primary (HN4, HN5, HN6, HN8, and HN10) and metastatic (HN1, HN2, HN3, HN7, and HN9) cell lines, when plotted separately, showed differences in both the amount of time for cytotoxic killing to occur, and in the variance of the distribution of survivabilities (Figure 3d). The metastatic cell lines were, in general, more resistant to immune‐mediated cytotoxicity than the primary cell lines with all metastatic cell lines exhibiting high survivability (>92%). In contrast, primary cell lines exhibited greater variability and earlier onset of response against NK‐mediated cytotoxic killing over the 24‐h duration of the assay.

**FIGURE 4:**
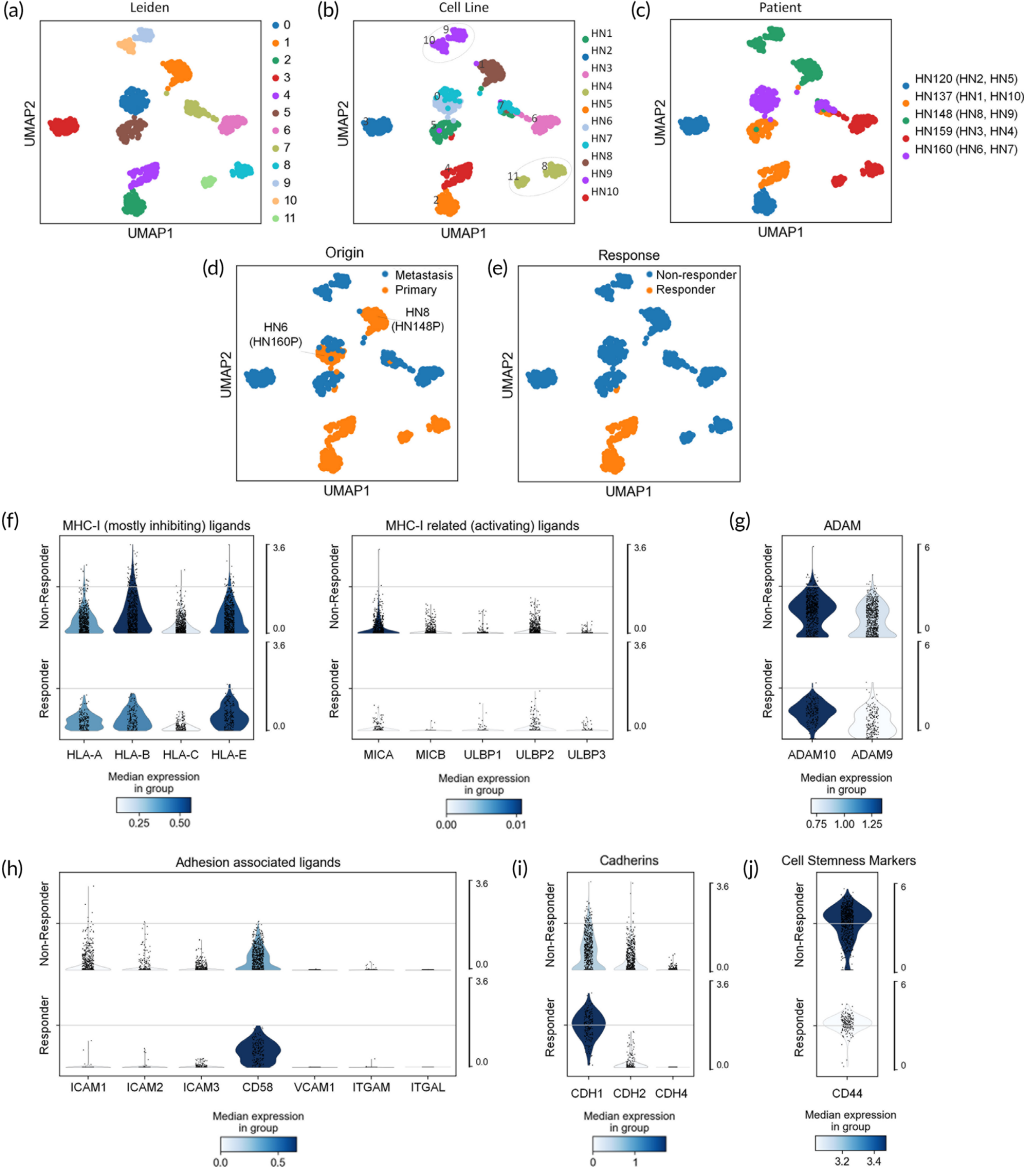
Downstream analysis and gene expression of key ligands found within the head and neck squamous cell carcinoma (HNSCC) data set grouped according to function. Downstream was performed using Scanpy (v. 1.4). (a) The Leiden algorithm was used for clustering and 12 unique clusters were identified and labeled on 2D UMAPs based on (b) cell line, (c) patient origin, (d) location of origin (primary or metastatic site) and (e) phenotypic response. (f) MHC‐I ligands were, in general, highly expressed among all cell lines (nonresponders and responders) but higher expression levels, especially of HLA‐B and HLA‐E were observed in nonresponder cell lines, contributing mostly inhibitory signals to natural killer (NK)‐92 cells. MHC‐I related (activating) ligands and other activating ligands exhibited very low expression levels with the exception of MICA (for nonresponders). ULPBs were higher among responders. (g) Increased ADAM 9/10 expression, which mediates cleavage of NKG2D ligands MICA/B from the tumor surface, was observed across all cell lines, and was higher in nonresponders. (h) Adhesion associated ligand CD58 was highly expressed in all cell lines but was higher in responders suggesting a more efficient binding to NK cells. (i) The epithelial marker E‐cadherin was more highly expressed in responders compared to nonresponders. Other cadherins (−N, −R) were moderately expressed or showed insignificant expression levels. (j) CD44, an HNSCC cell stemness marker often associated with poor prognosis was highly expressed in nonresponders.

Next, we sought an explanation for the differences observed between different cell lines and their origin based on their cell‐intrinsic gene expression signatures. We determined whether there were any correlations that existed between the on‐chip cell line response data and downstream transcriptomic analysis, at the resolution of single cells. The single cell RNA‐seq data sets are available in the Gene Expression Omnibus (GEO) repository under the accession number GSE117872. Differential gene expression analysis was conducted independently on all 10 HNSCC cell lines using single‐cell RNA‐seq (scRNA‐seq). Single cell libraries were first filtered (number of genes by total counts <10,000, percent mitochondrial genes <20%) normalized, and logarithmized, and highly variable genes were selected prior to performing principal component analysis (PCA) (Supplementary Data S1). A neighborhood graph of cells was calculated using best matched k‐nearest neighbor (KNN) to compute the optimal Uniform Manifold Approximation and Projection (UMAP) topology for dimensionality reduction. The use of Leiden‐based clustering algorithm^30^ identified 12 distinct clusters of cells (Figure 4a). Leiden clusters closely resembled the patient‐derived cell lines, with the exceptions of HN4 and HN9 that occupied two clusters each (clusters 8/11 and clusters 9/10, respectively) (Figure 4b). Most of the 10 cell lines examined exhibited unique clustering patterns except for HN6 and HN7 which co‐occupied cluster 0. This close clustering pattern is explained when the clusters are tagged according to patient identities as patient derived cell lines used in this study were obtained from 5 unique patients: HN120, HN137, HN148, HN159, and HN160 (Figure 4c). Cell lines HN6 and HN7, which co‐occupied cluster 0, originated from the same patient (HN160) explaining the clustering proximity, although all other primary/metastatic pairs from other patients exhibited separate clustering patterns, likely defined by their individual genomic/mutational signatures. Cluster labeling according to tumor origin (primary or metastatic) is shown in Figure 4d. HN6 (HN160P) and HN8 (HN148P) clustered more closely together with their metastatic counterparts and this is supported by the on‐chip cell line response data; HN8 and HN6 were the most resistant to NK‐mediated cytotoxicity among the primary cell lines at the E:T = 1:1 ratio. Labeling clusters according to on‐chip cell line response were also consistent with downstream analysis with the responders closely grouped toward the bottom of UMAP plot separate from nonresponders (Figure 4e) Thus, the response‐based stratification closely resembles the cell line stratification in the PCA space based on scRNA‐seq and hence correlates with transcriptomic and phenotypic profiles.

### 2.5 NK surface receptor activation/inhibition effect on cell line response

NK‐92 mediated cell cytotoxicity usually occurs via the release of cytolytic/cytotoxic granzymes and perforins. However, the degree of NK‐mediated cytotoxicity depends on the interplay and balance between the expression of distinct inhibitory and activating receptors. Extensive characterization of the known natural killer inhibitory and activating (NKARs) receptors involved in the cytolytic activity for different NK cell populations and their respective ligands is available in the literature.^31^, ^32^, ^33^, ^34^, ^35^, ^36^, ^37^ Metastatic cell lines are often more resistant to immune‐mediated killing and evasion from innate immunity due to associated (but not limited to) downregulation of activating ligands and/or upregulation of inhibiting ligands. However, based on our observed phenotypic responses of the 10 HNSCC lines, there were several exceptions to this trend (Figure 3c). Cell lines HN8 (148P), and to some extent HN4 (159P) and HN6 (160P), displayed strong to moderate resistance to NK‐mediated killing despite their primary tumor origin. In the case of HN 8, very little cytotoxic killing was observed. Thus, cell line origin—primary or metastatic—was not sufficient to determine response to NK‐mediated cytotoxicity.

We therefore considered the unique set of receptors/ligand pairs on the surface of NK‐92 cells (Table 2, Supplementary Data Table S4) and identified which of these pairs could potentially be the most important in terms of driving immune evasion mechanisms. NK‐92 cells are an immature form of NK cells that possess various MHC‐I inhibiting receptors and lack an important family of MHC‐I activating killer Ig‐like receptors (KIRs). This observation led to the hypothesis that regulation of MHC‐I inhibiting ligands may be at least part of the reason why the majority of metastatic HNSCC cell lines and some primary cell lines were generally more resistant to NK‐mediated cytotoxicity than their counterparts.

**TABLE 2:** NK‐92 receptor‐ligand pairs involved in NK cytotoxic activation/inhibition.

NK receptor	Alias	Ligands	In HNSCC data set	Exp. level	Ref.
*Inhibiting*					
ILT2	CD85j, LIR‐1	MHC‐1	HLA‐A/B/C	High	31, 35, 36, 38
KIR2DL2	CD158b1, NKAT‐6	MHC‐1/HLA‐A/C	HLA‐A/C	High	38, 39
KIR2DL3	CD158b2, NKAT‐2	MHC‐1/HLA‐C	HLA‐C	High	31, 35, 38
NKG2A/B/KLRD1	CD159a/CD94	MHC‐1/HLA‐E	HLA‐E	High	32, 33, 36
KLRG1	CLEC15A, 2F1, MAFA	E‐, N‐, R‐cadherins	CDH1/2,4	Moderate to high	35, 40
TIGIT	None	PVR (CD155)	PVR	Very low	31, 35, 38, 41
LLT1	CLEC2D	KLRB1 (CD161)/NKRP1A	KLRB1	None	42
FasL	CD178, FASLG	FAS (CD95)	FAS	Moderate	36
TRAIL	CD253, TNFSF10	DR4 (CD261), DR5 (CD262)	DR4/5 (TNFRSF10A/B)	Moderate to high	36
TWEAK	TNFSF12	DR3 (TRAMP)	DR3 (TNFRSF25)	Very low	36
TNF‐α	TNF, TNFSF2	TNFR1/2 (CD120a/b)	TNFRSF1A/B	Low to moderate	36
*Activating*					
NKG2E	KLRC3	MHC‐1/HLA‐E	HLA‐E	High	35, 36
LFA‐2	CD2, SRBC	CD58, CD59, CD15	CD58	High	31, 35, 36
NKp30	CD337, LY117, NCR3	HS GAGs, B7‐H6, Galectin‐3, BAG6	BAG6, NCR3LG1, LGALS3	Moderate to high	31, 35, 36, 39
NKG2D	CD314, KLRK1	MICA/B, ULBPs	MICA/B, ULBP1/2/3	Moderate	31, 35, 36
LFA‐1	CD11a (αLβ2 integrin)	ICAM‐1/2/3	ICAM‐1/2/3	Low to moderate	35, 36
NKp46	CD335, LY94, NCR1	HS GAGs, CFP, HA, PfEMP1	CFP, HSPG2	Very low	31, 32, 35, 36
VLA‐4 α chain	CD49d (α4 integrin)	VCAM‐1	VCAM‐1	Very low	35, 36
ICAM1	CD54	LFA‐1, MAC‐1	ITGAM, ITGAL	Very low	32, 36
TACTILE	CD96	PVR (CD155), Nectin‐2	PVR	Very low	31, 32, 34, 35, 38
2B4	CD244, SLAMF4, NAIL	CD48	CD48	None	31, 35, 36
*Both*					
KIR2DL4	CD158d, G9P	MHC‐1/HLA‐G	HLA‐G	Very low	36

Abbreviations: HNSCC, head and neck squamous cell carcinoma; NK, natural killer.

We attempted to reference the single‐cell transcriptomic data to identify key differences between the expression levels of genes that may be involved in determining the innate cytolytic response identified using the microfluidic platform (Figure 4f–j). DEG analysis identified genes expressed by the HNSCC cell lines that encoded known ligands for both activating and inhibiting receptors on NK cells. Intriguingly, we observed higher expression of classical (HLA‐A/B/C) and the non‐classical MHC class I ligands (HLA‐E) among the nonresponders compared to responders (Figure 4f, left panel). This suggested that upregulation of MHC‐I and HLA‐E was driving NK cell inhibition, which has been shown previously in other cancer types as providing a means of escape from NK surveillance.^34^, ^43^, ^44^, ^45^ In contrast, the expression levels of ULBPs (ULBP1/2/3) which bind to the activating KLR receptor NKG2D, were higher in responders compared to nonresponders (Figure 4f, right panel). However, expression levels for MICA/B, activating ligands for NK cells, were unexpectedly higher in nonresponders compared to responders (Figure 4f, right panel). This can be explained by the higher expression levels in nonresponders of ADAM, a disintegrin and metalloproteinase domain‐containing protein which mediates the cleavage of activating ligands MICA/B (Figure 4g). These soluble ligands can bind to the NKG2D receptor on NK‐92 cells acting as “molecular decoys” triggering NKAR downregulation.^34^, ^43^, ^46^, ^47^, ^48^ Expression levels of non‐MHC‐I or non‐MHC‐I related ligands, however, were relatively low (Supplementary Figure S7a) and their impact on the overall response of each cell line was considered minimal.^49^, ^50^, ^51^ More information regarding the single cell library expression data can be found in supplementary data Table S2.

Adhesion molecules also potentially play an important role in determination of response. Here we observed high expression levels of CD58 in all HNSCC cell lines but higher absolute and median expression levels in responders (Figure 4h). CD58 binds to the highly expressed LFA‐2 (CD2) receptor on NK‐92 cells. We speculate that responders exhibit more efficient binding with an ubiquitous receptor (CD2) on NK‐92 than nonresponders.

Interestingly, E‐cadherin, also a marker for differentiated epithelial cells, was expressed at significant levels across all cell lines but was enriched in responders compared to nonresponders (Figure 4i). Other cancer types such as triple negative breast cancer cell lines resistant to NK‐mediated cytotoxicity have been shown to exhibit decreased levels of E‐cadherin and undifferentiated, stem‐like properties that enable their escape from innate immune surveillance.^43^ Thus, we also examined expression levels of HNSCC specific cancer stem cell (CSC) markers, CD44 and ALDH, as well as other markers for HNSCC stemness, SOX2, NANOG, and BMI1 (Supplementary Figure S7b). These genes have been identified in the literature as having increased tumorigenicity and resistance to therapy^49^, ^50^, ^51^ (34–36). Most of the markers exhibited low expression levels across all HNSCC cell lines (Supplementary Figure S7b); however, CD44 expression levels were markedly different. Although all HNSCC lines expressed CD44, the nonresponders exhibited much higher expression compared to responders (Figure 4j). CSCs have been shown to be generally more resistant to therapy and our results support the idea that CD44+ non‐responding HNSCC cell lines may also exhibit enhanced resistance to immune mediated killing, thereby increasing their chances of evading the innate immune surveillance, and overall favor their chances of metastatic dissemination.

Finally, metastatic evasion of innate immunity can also occur by downregulation of the TNF death domain receptors. We examined four different ligand/receptor pairs (TNF‐α/TNF, FasL/FASLG/CD178, TRAIL/TNFSF10/CD253, and TWEAK/TNFSF12) but found that expression levels were not significant enough to highly impact response (Supplementary Figure S7c).

## 3 DISCUSSION

### 3.1 Adapting in vitro experiments for next generation solutions for immunotherapy

**FIGURE 5:**
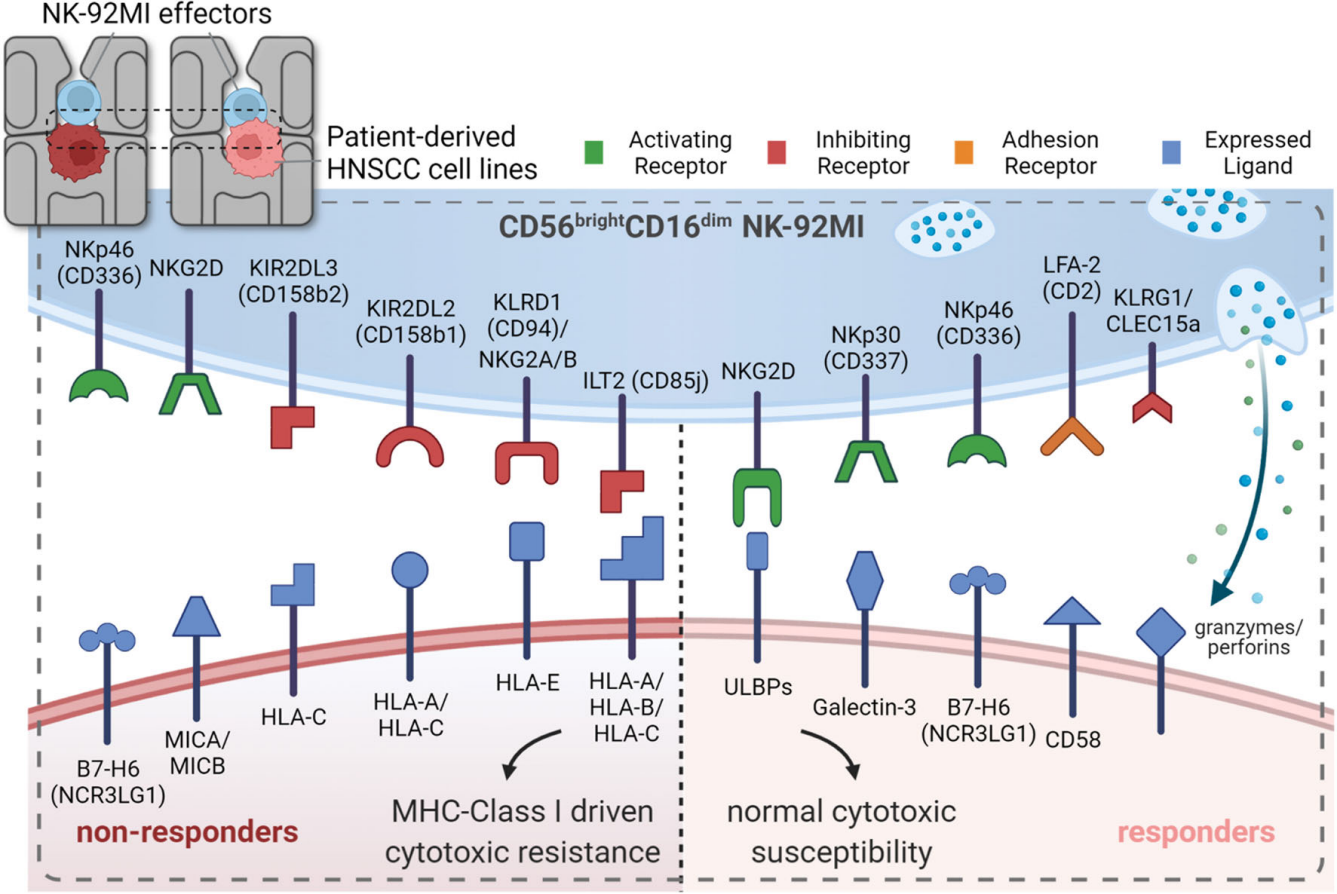
MHC‐I‐driven cytotoxic resistance observed in head and neck squamous cell carcinoma (HNSCC) nonresponder cell lines. Different combinations of signals from activating, inhibiting, and adhesion‐associated receptors interacting with specific ligands on the cell surfaces of different HNSCC cell lines resulted in differences in observed phenotypic response. Nonresponder cell lines exhibited higher expression levels of MHC‐I, MICA/B, and BAG6 ligands than responder cell lines while responder cell lines exhibited higher expression of ULBPs and B7‐H6, and moderate (although lower than nonresponders) expression of Galectin‐3. Responders also exhibited high expression of the binding‐associated ligand CD58 suggesting better binding efficiency than nonresponders and high expression of cadherins E‐ and N‐. Inhibition signaling and the establishment of a “pseudo‐self” state from MHC‐I binding was a dominant factor in determining differences in phenotypic response observed between nonresponders and responders as the number of inhibiting receptors present on natural killer (NK)‐92 cells: ILT2 (CD85j), NKG2A/KLRD1 (CD94), NKG2B/KLRD1 (CD94), KIR2DL2 (CD158b1), and KIR2DL3 (CD158b2) greatly outnumbered activating receptors (NKG2E). Although nonresponders exhibited higher values of the activating ligands MICA/B, they also exhibited high expression of ADAM10/9 which mediates cleavage of these ligands from the tumor surface. Inhibition signaling from ligands present on responder cell lines, including those coming from high expression of cadherins, were not as influential as signaling from MHC‐I and the binding‐associated ligand CD58, which binds to the ubiquitously expressed CD2 on the NK‐92 surface.

In this study, we present a new data‐driven phenotypic screening platform, which combines a microfluidic cell‐trapping device with a DL computer vision analysis program to generate a massive amount of cell interaction data between 10 different patient‐derived HNSCC cell lines and NK92‐MI effector cells. We were thus able to employ statistical modeling to compare the differential responses of these different tumor cell lines to NK92‐MI‐mediated cytotoxicity in an in vitro on‐chip assay with single cell resolution and identify trends and outliers among the cell lines as well as define a group of responders and nonresponders. We then coupled this information with downstream transcriptomic analysis to explain how differences in key NK‐92 receptor ligand expression levels can influence response (Figure 5). Our results revealed that MHC‐I driven cytotoxic resistance played the most significant role in determining differential response, and this result is consistent with established models in the literature.^34^, ^45^ Furthermore, we attempt to demonstrate that adaption of next generation solutions for in vitro immunotherapy experimentation can greatly benefit from the combination of microfluidic platforms with automated computer vision and downstream transcriptomic analysis. Here, we were able to effectively exploit the specific advantages of microfluidic cell trapping devices to rapidly observe differences in phenotypic response among different HNSCC cell lines using *accurate* E:T ratios and observe and measure the outcomes of those interactions longitudinally. Our approach has several advantages over conventional coculture models, which either do not control the interacting cells at all (e.g., random coculture in well plate) or compartmentalize tumor and immune cells at the population level (e.g., transwell). These include more precise control over large number of tumor‐immune interacting partners via microfluidic cell traps, and the fast and automatic analysis of massively standardized data sets, which are amenable to comparison and ad hoc querying post‐experiment.

Our motivation for advocating for transitioning from traditional population‐based coculture systems (e.g., well‐plates) to microfluidic‐based in vitro testing platforms is driven by the view that they are best suited to accommodate accurate and statistically relevant high‐throughput tumor‐immune models that can aid in the detection of heterogeneity among patients as well as the standardization of immune assessment and immune response as an important part of diagnosis and the HNSCC grading system for clinical trials.^5^ Despite being one of the most common and ubiquitously used methods for coculture of effector and target cells together in vitro, well plates still have unique challenges which can limit the reliability and accuracy of the intended cytotoxicity model.^22^ Creating controlled and regular cellular interactions in well plates can be quite challenging^52^ and bulk 2D cell culture is often prone to localized, variable E:T ratios that can widely differ from the intended E:T ratio (Supplementary Figure S1a left panel). Analysis of these interactions often requires the selection of ROIs within the well plates. However, drawing meaningful boundaries which define the ROIs in 2D cell culture can be arbitrary and variable (Supplementary Figure S1a right panel) as well as very time‐consuming and can potentially lead to reproducibility issues and differences in experimental results across platforms (Supplementary Figure S1b,c). For time‐series experiments or experiments requiring the tracking of specific cells, the well‐plate format is also not ideal; the positioning of non‐adherent cells in bulk solution tends to change gradually over time and unless continuous live‐cell monitoring is conducted it can be difficult to locate and identify individual cellular interactions.

By using the microfluidic trapping device for conducting tumor‐immune interaction assays, we could address the drawbacks of using well plates for bulk cell culture discussed previously, as well as strengthen the case for using microfluidic‐based in vitro testing platforms by introducing an automation element into the analysis process. This was possible because of the fixed positioning of the cell traps which define the ROIs in which cellular interactions are potentially taking place, in both space and time (Figure 1c). Although the unique designs of many microfluidic platforms often require manual analysis of image output, the automated DL computer vision algorithm which we deployed allowed us to avoid the arduous task of having to manually inspect hundreds of thousands of individual cell trap images. Since these algorithms and neural networks are becoming much easier to deploy and apply to different applications,^53^, ^54^, ^55^, ^56^ we also predict the use of DL computer vision to become standard practice for analyzing live‐cell imaging data in the future especially within multiplexed, or high N microfluidic devices.

Limitations to this particular experimental approach include an inability to retrieve and analyze individual cells within particular cell traps to observe changes in expression levels before and after pairing. The data presented here thus represents a single time point in expression level data. In future work, we plan to address these issues to improve the complexity of the NK‐tumor models that include other cells of the TME, such as MDSCs, CAFs, endothelial cells, and enable recovery of both effector and target cells post interaction on‐chip.

## 4 CONCLUSIONS

Overall, our integration of an in vitro platform using a microfluidic chip‐based approach to conduct TIDI experiments, and a unique method for the creation and management of large, statistically relevant data sets of phenotypical cell interaction behavior, coupled with transcriptomic sequencing data on the single‐cell level provides a strong alternative method for detection of patient‐specific heterogeneity and can potentially act as a tool for accelerating biomarker discovery and combinatorial immunotherapy testing.

## 5 MATERIALS AND METHODS

### 5.1 Experimental design

Our primary objective was to use a controlled and accurate microfluidic immune‐tumor pairing cytotoxicity assay to identify phenotypic differences among patient‐derived HNSCC cell lines, which could then be compared to transcriptomic expression profiles to determine potential causes for heterogeneity of patient cell line response. We designed our experimental workflow to incorporate three key components: an in vitro microfluidic coculture cell pairing assay, DL computer vision image analysis to quickly track, identify and quantify cytotoxicity, and single‐cell RNAseq analysis to determine difference in expression profiles between patient derived cell lines which resisted or responded to NK‐mediated killing.

### 5.2 PDC cell lines

PDC head and neck squamous cell carcinoma (HNSCC) cell lines HN120, HN137, HN148, HN159, and HN160 comprised of both primary and metastatic tumors from HNSCC patients were obtained from our previous derivation protocol.^10^ Briefly, tumor samples from patients were minced and subsequently disassociated using 4 mg/ml of collagenase type IV (Thermo Fisher) in DMEM/F12 (Thermo Fisher) at 37°C for 2 h. Cells were washed, pelleted, and resuspended in phosphate‐buffered saline (PBS; Thermo Fisher) for three cycles and finally strained using a 70 μm cell strainer before pelleting and resuspension in RPMI (Thermo Fisher).

### 5.3 Cell Culture

All HNSCC cell lines were cultured and expanded in T‐25 flasks (ThermoFisher) using RPMI 1640 medium enriched with 10% Fetal Bovine Serum (HyClone, USA) and 1% Penicillin Streptomycin (ThermoFisher). Cells were maintained in a humidified atmosphere of 5% CO_2_ at 37°C. Medium was changed every 2–3 days and the cells were passaged when reaching about 80% of confluency (~every 3 days). NK92MI cells (ATCC, USA) were cultured and expanded in T‐25 flasks (ThermoFisher) using alpha‐Minimum Essentials Eagle Medium (MEM) enriched with 12.5% FBS (HyClone, USA), 12% horse serum (ThermoFisher), 4% MEM vitamin solution (ThermoFisher) and 0.1 mM beta‐mercaptoethanol (Thermofisher).

### 5.4 Microfluidic device and fabrication

The cell pairing devices are fabricated using polydimethylsiloxane (PDMS) soft lithography. Devices were molded on polyurethane master molds replicated from the photoresist SU‐8 wafers. The PDMS devices were prepared from a 10:1 ratio suspension of base and curing agent (Sylgard 184, Dow Chemical), degassed for 1 h and then cured for 90 min at 70°C. Access ports at the inlet and outlet were made using a 1.5 diameter biopsy punch (Miltex, 15110‐15). The devices were then cleaned to remove any debris and plasma bonded (Pie Scientific) to 75 × 25 × 1 mm glass coverslips. Finally, the devices were autoclaved for 2 h and left to dry overnight at 50°C. Before usage, each microfluidic PDMS device was coated with 2% Pluronic acid solution mixed with 1X PBS for 2 h under a BSC, washed twice PBS and primed with cell culture medium.

### 5.5 Cell staining

HNSCC and NK‐92MI cells suspensions were harvested and stained with 5uM CellTracker Orange CMTMR dye (Thermofisher) in PBS and 10uM CellTracker Violet BMQC in PBS for 10 min, respectively. The cells were then washed in PBS twice prior to resuspension at a concentration of 2 M cells/ml in their respective media.

### 5.6 2D well‐plate coculture and end‐point viability assays

HNSCC cell lines were seeded at 104 cells/well and allowed to attach to the bottom of the well overnight. The cells were then stained with CellTrackerTM Orange (Thermo Fisher Scientific, C2927) and washed, and a suspension of NK‐92MI cells were added to the wells at 1:1, 2:1, 3:1, and 10:1 effector‐tumor (E:T) ratios (*n* = 3) along with a no‐NK cells control group. Cell medium containing 4 μM concentration of Cell EventTM Caspase‐3/7 apoptosis green detection agent was added to each of the wells to maintain a constant 0.1 ml volume, and the coculture was allowed to incubate overnight using live‐image microscopy to capture images every 8 h using a high‐content analysis imaging platform. The images, capturing an area of 2675.5 μm^2^ in each well, were analyzed using ImageJ to calculate cell viability which was averaged (*n* = 3) at each time point for all four cell lines (Figure 1b). End‐point viability measurements were quantified at 24 h for all four cell lines at each of the different E:T ratios.

### 5.7 Microfluidic coculture assay

For the microfluidic assay a P‐200 ETFE female ferrule (IDEX Health and Science LLC, USA) was attached to one side of a cut Teflon PFA resin (PFA) tubing (IDEX Health and Science LLC), followed by the attachment of one XP‐235 PEEK nut (IDEX Health and Science LLC) and one P‐658 female Luer (IDEX Health and Science LLC). These fittings were used to connect to a 5 ml syringe containing cell medium while the other free end of the tubing was connected to the inlet of the cell pairing microfluidic device. A short PFA tubing was connected to the outlet of the device at one end and taped to a 15 ml conical tube for waste collection at the other. The syringe was then connected to a syringe pump (KD Scientific, USA, Legato 111) and the device was placed on the platform of a microscope (Leica Microsystems, Germany, DMi1) inside a laminar hood cabinet. The microfluidic devices were primed with 0.2% bovine serum albumin (Millipore) diluted in PBS for 2 h and degassed. The devices were infused with the Caspase‐free seeding medium. Then, 2 μl of a previously prepared tumor cell suspension was then added into the inlet of the devices which was then connected to the syringe pump. The initial flow rate was set for 0.1 ml/h to allow the cells to enter the trap chamber and position themselves at the inlet of each trap. The flow was then gradually increased up to 1 ml/h to remove all the cells at the inlet of the device and then raised up to 5 ml/h to push the HN cells into each chamber while washing away the residual cells. This procedure ensured that more than 90% of the cell traps contained at least one HN cell. After the cancer cell seeding, a similar procedure was used for seeding the NK cells. The syringe pump tubing was disconnected and 5 μl of NK cell suspension were pipetted into the inlet of the device. The tubing of the syringe pump was again connected and an initial flow rate of 0.1 ml/h was set to allow the immune cells to flow into the cell traps region. Gradually, the flow rate was raised up to 5 ml/h to push the NK cells into the traps and wash the leftover away from the device. Once both cell types were seeded, the device was ready for 24 h live‐cell imaging.

### 5.8 Live cell imaging

Here, 24‐h live imaging was conducted via perfusion of the microfluidic cell trapping device. Media used for the 24 h experiment was generated by adding 60 mM Hepes buffer solution (Thermofisher) to a mix of HN and NK medium (volumetric ratio = 1:1). Then, 4 μM of CellEvent Caspase‐3/7 Green Detection Reagent (ThermoFisher) was added to monitor the death rate in situ. A 5 ml syringe was filled with cell medium and connected to a long PFA tubing that replaced the tubing used for cell seeding. The tubing used at the outlet of the microfluidic chip was reused. The device was brought and secured on the platform inside an incubation chamber for live cell imaging on a LSM800 Confocal microscope. The syringe was connected to a syringe pump and the flow rate was set at 0.01 ml/h during the continuous acquisition. The imaging software (Zen, Zeiss) was set to include the whole cell trap region into the device with a single 2D scan via merging and stitching of multiple tiles. One track was set for each dye to best fit the signal and avoid cross talk between the fluorescent channels. Each acquisition was taken every 20 min to ensure adequate time resolution for detection of cellular interaction events.

### 5.9 CNN design and conversion to R‐CNN for object detection

CNNs were created using MATLAB (R2021a). The CNNs were based off the architecture of resnet‐50 and imported using the built‐in import command *resnet50*(). In following of the transfer learning protocol, for RCNN‐1, the last three layers of the network were removed and replaced with two branches; the first branch contained new image classification layers: a fully connected layer (RCNN‐1: number of outputs = 2, one class + background, RCNN‐2: number of outputs = 5, four classes + background), a softmax layer and a classification layer while the second branch contained a fully connected layer (number of outputs = 4) and a box regression layer (Supplementary Figure S3). Several modifications were also added between convolution blocks 4 and 5 of the resnet‐50 network to create a regional proposal network. A convolutional 2D layer followed by a rectified linear units (ReLu) layer was added immediately following convolution block 4. The ReLu layer output was connected to (1) the region proposal classification output layers comprised of a 2D convolution layer, a softmax layer, and a regional proposal classification layer (number of outputs = 2) as well as (2) the region proposal box regression output layers comprised of a 2D convolution layer and a RCNN box regression layer (number of outputs = 4) to predict box offsets. The convolution layers of region proposal classification and the region proposal box regression were both inputs to a region proposal layer (RCNN‐1: 1 anchor box, size = [120100], RCNN‐2: 1 anchor box, size = [16 16]) which was followed by a ROI 2D max pooling layer (output size = [14 14]). The output of this max pooling layer was input to convolution block 5.

### 5.10 Training image data set preparation

Two different data sets were used for neural network training. RCNN‐1 was trained using a data set comprised of >1000 pixel‐labeled images (691 × 751 pixels) which were cropped from the stitched bright‐field image data set. These images were generated using a separate code written in the MATLAB appDesigner development environment. The app created in appDesigner allowed for the quick labeling of trap objects using template searching (Supplementary Movie S4). After labeling, the images were saved and compiled into a training data set which was augmented using random reflections (*randomAffine2d*) in the x dimension. RCNN‐2 was trained using a data set comprised of >3000 pixel‐labeled images (56 × 72 pixels) that were cropped from the full stitched RBG images of the device using ROI information generated from the analysis of the trap positions using RCNN‐1. These images were labeled according to the position and status of the cells and saved using the native MATLAB image labeler application. Prior to training, the data set for RCNN‐2 was also augmented in a similar manner using transformation via random reflections in the x dimension.

### 5.11 R‐CNN training and testing

RCNN‐1 was trained using the built‐in *trainFasterRCNNObjectDetector* function in MATLAB. The command required 5 inputs, including the training data set, the network to be trained (RCNN‐1), training options, and a negative overlap and positive overlap range for determining successful object identification. The training options involved setting the training algorithm (solver) to stochastic gradient descent with momentum for updating the network parameters (weights and biases) to minimize the loss function. The initial learn rate was set to 1 × 10^−3^ with a “piecewise” learn rate schedule. The learn rate drop factor was set to 0.1, and the learn rate drop period was set to 100. The maximum number of epochs for learning was set to 3 with a minibatch size set at 6 images. The negative overlap range was set between [0 0.3] while the positive overlap range was set at [0.5 1.0]. RCNN‐2 was also trained using the *trainFasterRCNNObjectDetector* function. The training options were identical to the training options for RCNN‐1 with the exception of the maximum number of epochs for training (=75), the minibatch size (=24 images), and the positive overlap range (= [0.6 1.0]).

### 5.12 R‐CNN deployment and data set generation

RCNN‐1 was deployed using a separate program written using the built in appDesigner development environment to create an app which allowed for the visualization of the full microfluidic device (stitched image). The app takes as input a raw stitched bright‐field image data set comprised of ~72 images. The first image in the data set is displayed. The app creates a *N* × 3 (rows × columns) data structure—a cell array variable named *cellmatrix*—where *N* is the number of images in the data set. The *cellmatrix* structure contains the bounding box information, the confidence level scores, and the labels (i.e., “cell trap”) for all identified traps in the stitched image for all images in the data set. The data are recorded in the structure after iteratively analyzing each image in the data set using the built‐in MATLAB *detect* function designed for object detection networks. Detection was executed with a minibatch size = 1, number of strongest regions = 3000, a maximum objection detection size of 130 × 110 pixels and a minimum object detection size of 65 × 55 pixels and a threshold of 0.5. The data contained in the *cellmatrix* variable is processed using another separate code to order the traps and filter out any traps that were not able to be identified throughout the entire time series. After processing, trap position and numbering data are used to cut the full‐sized stitched image of the entire microfluidic chip into small (56 × 72 pixel) images which are ordered according to trap number and time. The first full stitched image in the time series is used to establish the E:T ratios (assumed constant). This is performed by first using the built in MATLAB function *imfindcircles* which uses a circular Hough transform based algorithm to detect circular objects in images. The E:T ratios and the ordered, cropped trap image data sets are then passed to another written function which, similarly to RCNN‐1, utilizes the built in MATLAB detect function (minibatch size = 1, number of strongest regions = 12, maximum object detection size = 96 × 96 pixels, minimum object detection size = 16 × 16, threshold = 0.99) to identify cell death events in the cell traps. Finally, the cell death events and the individual cell traps in which they occurred are tracked and recorded. RCNN‐2 outputs a spreadsheet of data containing information regarding the trap number, the time of cell death event (h), the status of each trap after 24 h (1 = death event occurred, 2 = no mediated cell death event), the E:T ratio population to which the trap belonged.

### 5.13 Single cell RNA‐Seq data

Single cell library data were obtained from GEO: GSE117872 and were originally generated using the following protocol: a C1 Single‐Cell Auto Prep IFC (Fluidigm) system was used to capture single cells. Reverse transcription and cDNA pre‐amplification was conducted on the C1 chips using the SMARTer PCR cDNA Synthesis kit (Clontech) and the Advantage 2 PCR kit (Clontech). The single cell libraries were established using the Nextera DNA Sample Preparation Kit and the Nextera Index Kit (Illumina).

### 5.14 Kaplan–Meier survivability

Kaplan–Meier survivability curves were generated using OriginPro (v2021b 64‐bit SR2). Spreadsheet formatted data generated by MATLAB was imported directly into the software and graphs were generated using the built‐in survival analysis Kaplan–Meier estimator tool. Normalized experimental survivabilities were calculated according to:

$$S_{\exp\_\mathrm{norm}}\left(t\right)=\frac{S_{\exp}\left(t\right)-\min\left(S_{\text{cont}}\left(t\right)\right)}{\max\left(S_{\text{cont}}\left(t\right)\right)-\min\left(S_{\text{cont}}\left(t\right)\right)}$$

All graphs and statistical calculations were generated using OriginPro (OriginLabs) software. Statistical significance was computed using survivability analysis and log‐rank test statistics or with the test indicated in each figure legend. The number of experiments performed and the number of repeat cellular interaction experiments in each representative population are indicated in each figure.

### 5.15 Single cell library preprocessing

Scanpy, a scalable Python‐based package (v 1.4) designed for single cell gene expression data sets was used for clustering and downstream analysis and coding was performed using Jupyter. Preprocessing of the single cell libraries involved filtering for cells expressing a minimum of 200 unique genes which are expressed by a minimum of 30 cells. Counts per each cell were normalized by the total counts over all genes using *scanpy.pp.normalize_total* with a scaling factor of 10,000 such that each cell had the same total count after normalization. Data were logarithmized using *scanpy.pp.log1p*. Highly variable genes (minimum dispersion = 0.5, 0.0125 ≤ mean ≤ 3) were defined and identified using *scanpy.pp.highly_variable_genes* and filtered out. Cells expressing more than 10,000 genes and dying cells with a mitochondrial gene expression percentage greater than 20% were regressed out with *scanpy.pp.regress_out* and data were scaled to unit variance with *scanpy.pp.scale*.

### 5.16 PCA and clustering, and downstream analysis

After preprocessing, PCA was conducted using *scanpy.tl.pca* with 50 PCs and ranking of variance ratios was visualized with s*canpy.pl.pca_variance_ratio*. To obtain better clustering, a neighborhood graph of cells was first calculated using *scanpy.pp.neighbors* (neighbors = 10, PCs = 50) and the KNN was automatically weighted by the algorithm to compute the optimal UMAP topology. The neighborhood graph was embedded into the UMAP using *scanpy.tl.umap* (minimum distance = 0.5) and the Leiden method (*scanpy.tl.leiden*, resolution = 1.0) was then used to determine the different clusters of cells. Clusters were labeled according to cell line, patient origin, site origin (primary or metastatic) and response (determined by the cell trapping in vitro killing assay and survivability analysis).

## AUTHOR CONTRIBUTIONS

**Christopher P. Tostado:** Conceptualization (equal); formal analysis (lead); funding acquisition (lead); investigation (supporting); methodology (equal); resources (supporting); visualization (lead); writing – original draft (lead); writing – review and editing (lead). **Lucas Xian Da Ong:** Data curation (supporting); investigation (equal). **Joel Jia Wei Heng:** Formal analysis (supporting); investigation (equal). **Carlo Miccolis:** Investigation (supporting); methodology (supporting); writing – original draft (supporting). **Shumei Chia:** Methodology (supporting). **Justine Jia Wen Seow:** Data curation (supporting); formal analysis (supporting); visualization (supporting). **Yi‐Chin Toh:** Conceptualization (equal); funding acquisition (supporting); resources (supporting); supervision (equal); writing – review and editing (supporting). **Ramanuj DasGupta:** Conceptualization (equal); funding acquisition (supporting); methodology (supporting); project administration (supporting); resources (lead); supervision (equal); validation (supporting); writing – review and editing (supporting).

## CONFLICT OF INTEREST STATEMENT

The authors declare no competing interests.

### PEER REVIEW

The peer review history for this article is available at https://www.webofscience.com/api/gateway/wos/peer-review/10.1002/btm2.10628.

## Supporting information


**DATA S1.** Supporting Information.

**TABLE S2.** Filtered, normalized, logarithmized, single cell library expression data related to Figure 4. This data set contains data for 1247 individual single cell libraries expressing 16,383 genes.

**TABLE S3.** Raw data set of outputs from R‐CNN2 related to Table 1 and Figure 3. This data set contains the results of the analysis of all 10 HNSCC cell lines' cell trap interaction data over the 24 hr on‐chip cell trapping experiments in excel spreadsheet format. The data set contains information regarding the trap number, the time of cell death event (hrs), the status of each trap after 24 hr (1 = death event occurred, 2 = no mediated cell death event), the population to which the trap belonged to (i.e., the E:T ratio).

**TABLE S4.** Expanded NK‐92 receptor‐ligand pair spreadsheet related to Table 2. This data set is the expanded version of Table 2 in the main text. The comprehensive list of NK‐92 receptors and corresponding ligands provides evidence for why key ligands present in the HNSCC single cell libraries were either considered or not considered when drawing conclusions regarding the behavior of non‐responsive or responsive cell lines.

**MOVIE S1.** NK‐92 mediated killing of HNSCC (E:T = 1:1) related to Figure 1e. A single NK‐92 cell with a single HNSCC tumor cell in forced close contact inside a microfluidic cell trap.

**MOVIE S2.** NK‐92 mediated killing of HNSCC (E:T = 2:1) related to Figure 1e. Two NK‐92 cell with a single HNSCC tumor cell in forced close contact inside a microfluidic cell trap.

**MOVIE S3.** NK‐92 mediated killing of HNSCC (E:T = 3:1) related to Figure 1e. Three NK‐92 cells with a single HNSCC tumor cell in forced close contact inside a microfluidic cell trap.

**MOVIE S4.** R‐CNN1 training data set generation related to Figure 2b “Pattern Recognition.” A graphical user interface was designed with a separate code using the MATLAB appDesigner development environment. This app was designed to integrate a MATLAB pattern recognition algorithm that could be used to quickly label and distinguish whole and partial traps within images to be used for ground truth training data.

## Data Availability

Data that support the findings of this study are openly available in the Gene Expression Omnibus (GEO) repository under the accession number GSE117872. Any additional data that support the findings of this study are available from the corresponding author upon reasonable request.
