# Supplementary material for: An AI‐assisted integrated, scalable, single‐cell phenomic‐transcriptomic platform to elucidate intratumor heterogeneity against immune response
Source: Bioeng Transl Med. 2024 Jan 2;9(2):e10628. doi: 10.1002/btm2.10628 (PMC10905538; doi:10.1002/btm2.10628)
Supplement: Supplementary file 1 — DATA S1. Supporting Information. [file BTM2-9-e10628-s002.docx]

**Supplementary Material**


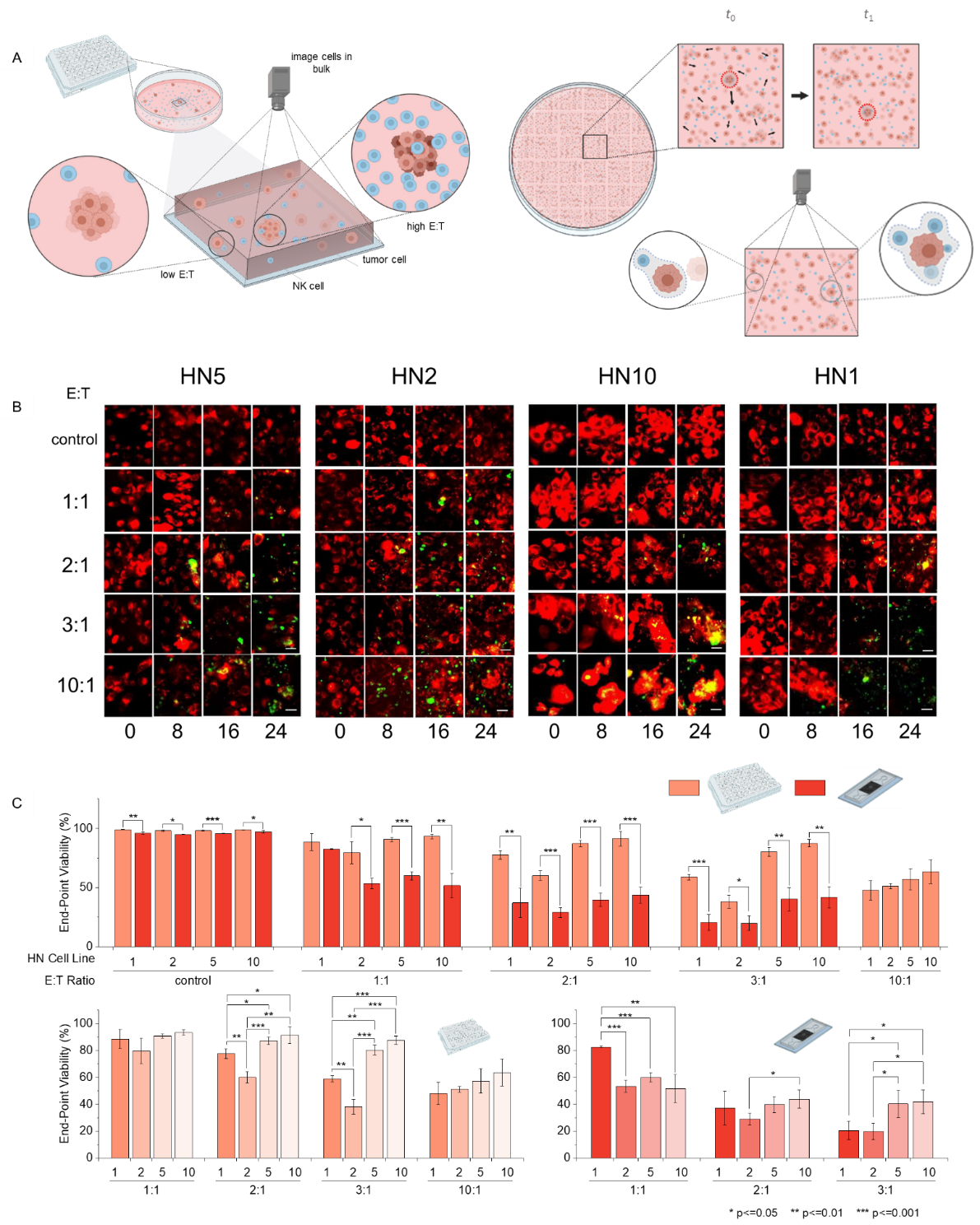


Fig. S1 Endpoint tumor-immune cytotoxicity assays conducted in well plates versus microfluidic cell pairing device related to Figure 1A,C.

**(A)** (left panel) Reproducibility and accuracy of the immune-tumor interaction models developed in well-plate format can highly vary due to localized, variable effector:target (E:T) ratios, difficulty with tracking specific cell interactions over time in a dynamic environment, and (right panel) arbitrarily defined regions-of-interest. **(B)** 2D well-plate co-culture experiments were conducted using four different HNSCC cell lines (stained red) cultured together with NK-92 cells at different E:T ratios for 24 h. Cell apoptosis was monitored using a caspase-3/7 apoptosis detection agent (green). In general, increased apoptosis was observed as time progressed at higher E:T ratios however higher amounts of apoptosis were observed at higher E:T ratios most likely due to differences in localized effective E:T ratios. At E:T = 1:1, only HN2 showed significant signs of apoptosis at t > 16 h. **(C)** Differences in tumor cell end-point viabilities observed between the 2D co-culture conducted in a traditional 96-well plate format and the co-culture conducted on-chip at different E:T ratios for four different HNSCC cell lines. Lower end-point viabilities were observed on-chip when compared to their well-plate counterparts at equivavlent E:T ratios. Two-tailed t-test p-values are shown.


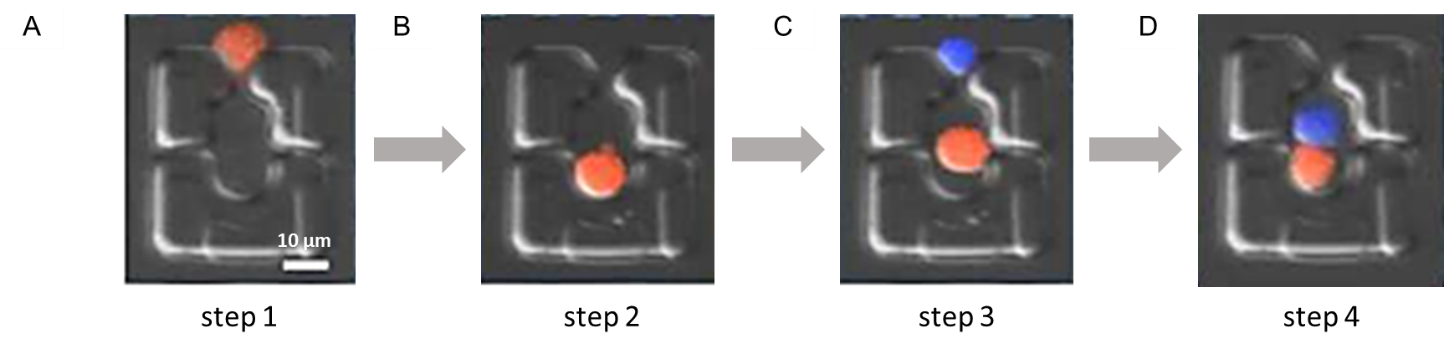


Fig. S2. Cell seeding process in the microfluidic trap structures related to Figure 1D,E.

Hydrodynamic seeding of cells was achieved using a four-step process. A) Singularized tumor cells were first seeded into the device at a low flow rate of 0.1 ul/hour. Hydrodynamic flow patterns position the cells at the opening of the microfluidic cell traps. Once most of the traps possessed a tumor cell at the opening, the flow rate was slowly increased to 1 mL/hour to remove any cells in the device not positioned at the opening of a trap. B) The flow rate was then raised to 5 mL/hour to push the cells at the opening of the traps into the trap. C) The same process was repeated for trapping the second cell type, NK-92 MI effector cells, into the devices. D) The cell seeding process results in a distributed population of effector:tumor ratios within the traps with a majority of traps possessing a 1:1 ratio.


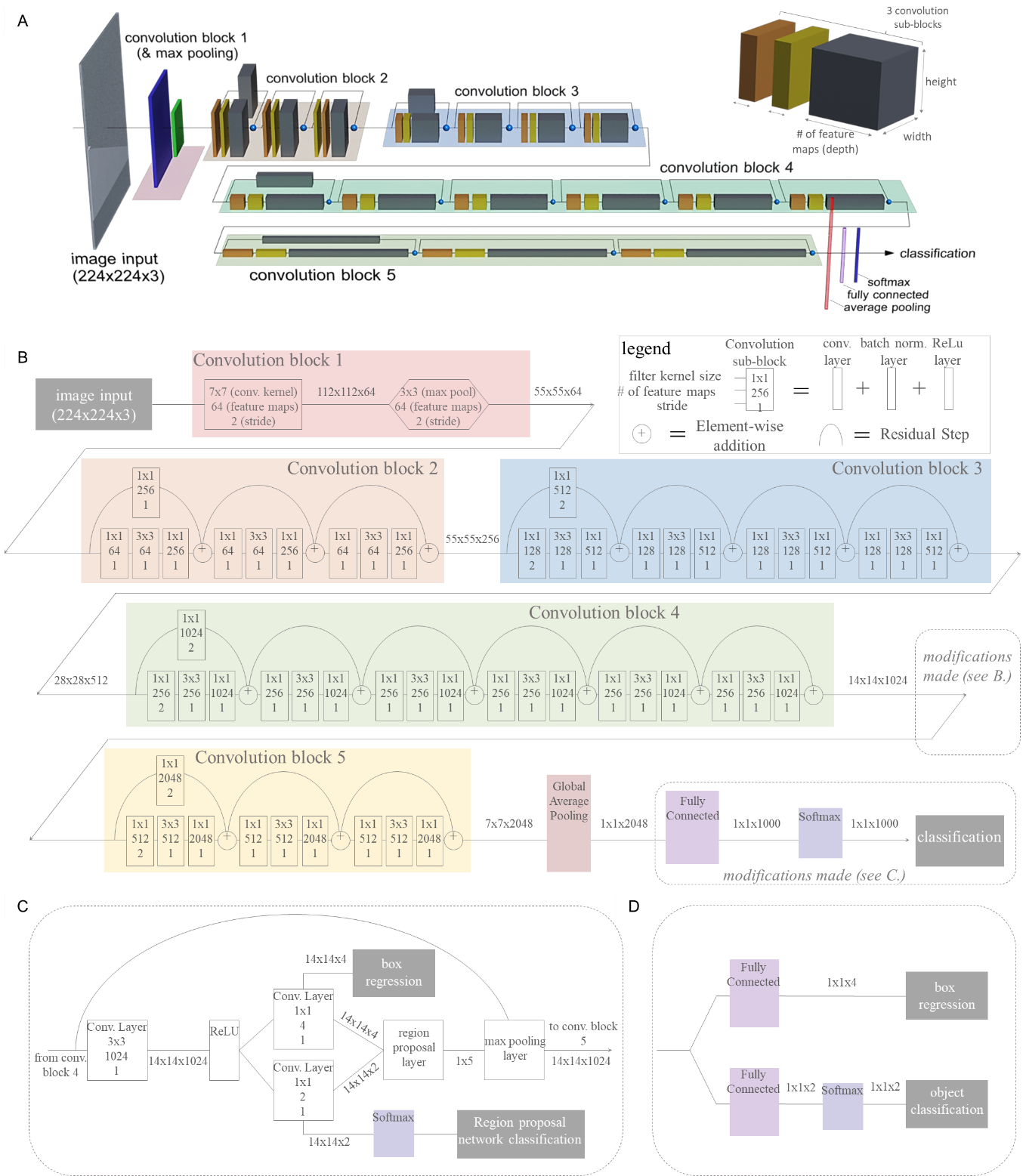


Fig. S3. Convolutional neural networks (CNNs) used for image analysis related to Figure 2.

Neural networks used for deep-learning image analysis were based on the architecture of Resnet-50, a residual network optimized for image analysis. A) 3D visualization of the Resnet-50 network. The network accepts a 224×224×3 pixel image as input, which is manipulated using convolution and max or average pooling steps to change its representational dimensions. The height and width of each block correspond to the height and width of the input at each layer while the depth corresponds to the number of feature maps describing the input. B) A detailed breakdown of the operations performed at each layer within the network. The network is comprised of five convolution blocks. Convolution Block 1 contains two layers. Convolution Blocks 2 through 5 contain three, four, six, and three residual blocks, respectively. Each residual block is comprised of 3 sub-layers: a convolution layer, a batch normalization layer, and a rectified linear units layer. The 5 convolution blocks comprise 50 (2+3×3+4×3+6×3+3×3) layers total. Residual networks allow for “short-circuiting” of the network; layers which are not useful for improving the accuracy of the network (by avoiding diminishing gradients during backpropagation) can be skipped. C) Conversion from a CNN to a regions with convolution neural network (R-CNN) for object detection involved modification of the Resnet-50 network. Additional layers were added between convolution blocks 4 and 5. D) A branch in the network prior to the last three operational layers was also required, allowing for optimization of the box regression used for determining the area (locations) for detected objects.


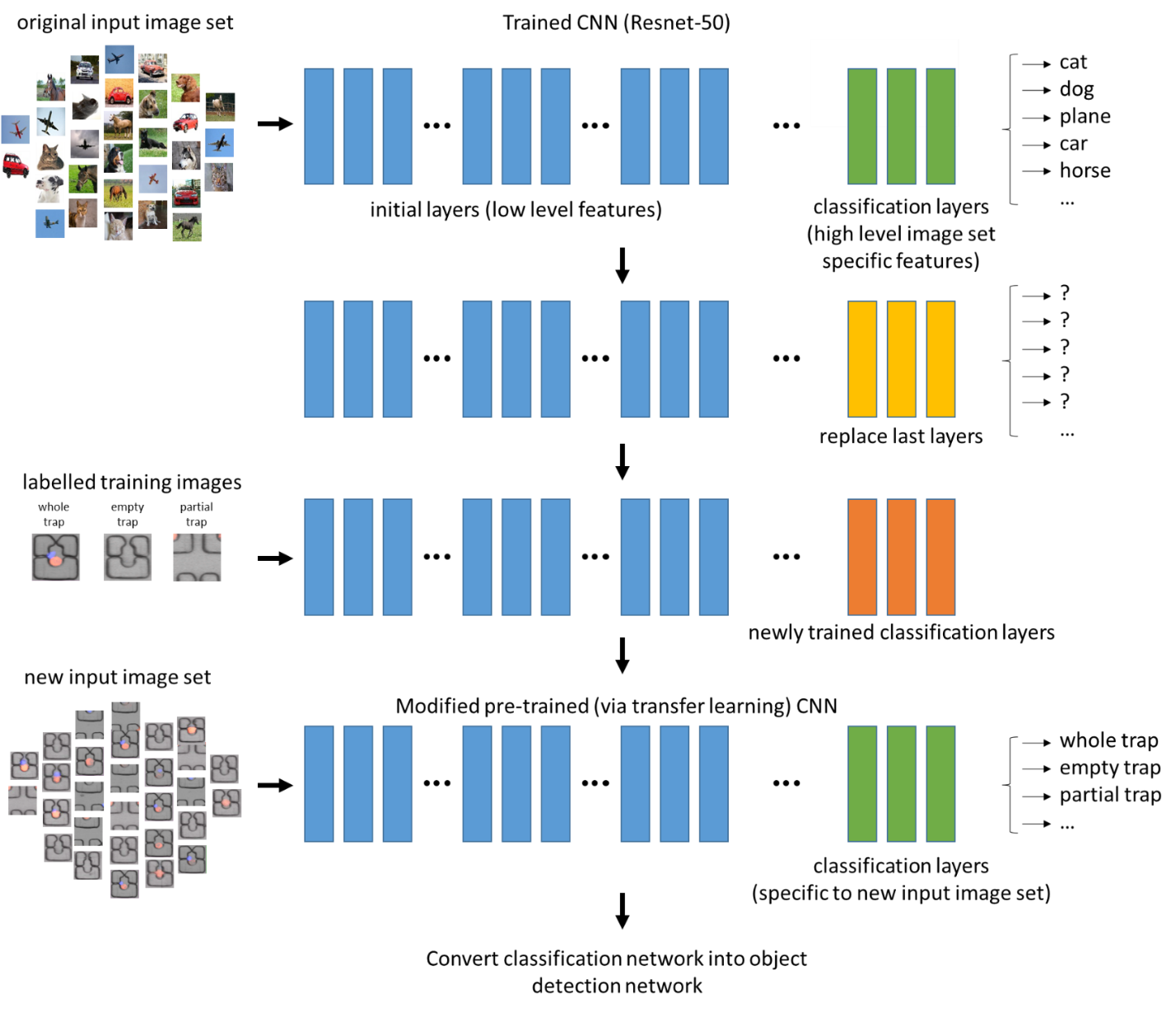


Fig. S4. The transfer learning process used for training the R-CNNs related to Figure 2.

The neural network used in this study, Resnet-50, is a convolutional neural network that has been trained on more than a million images from the ImageNet database (original labelled training set) for classifying images into object categories. Transfer learning takes advantage of the initial layers in Resnet-50 which have already been trained to recognize low-level features in images (such as edges or colors) and uses them in a new network in which the final classification layers have been removed and replaced with layers that can accommodate a different number of object classes. The new network can be re-trained by defining a new number of object classes and by using a new labelled training image set. After training, the new modified network can be used with a new image input set and the network can determine the image class based on the new number of object classes and limited training.


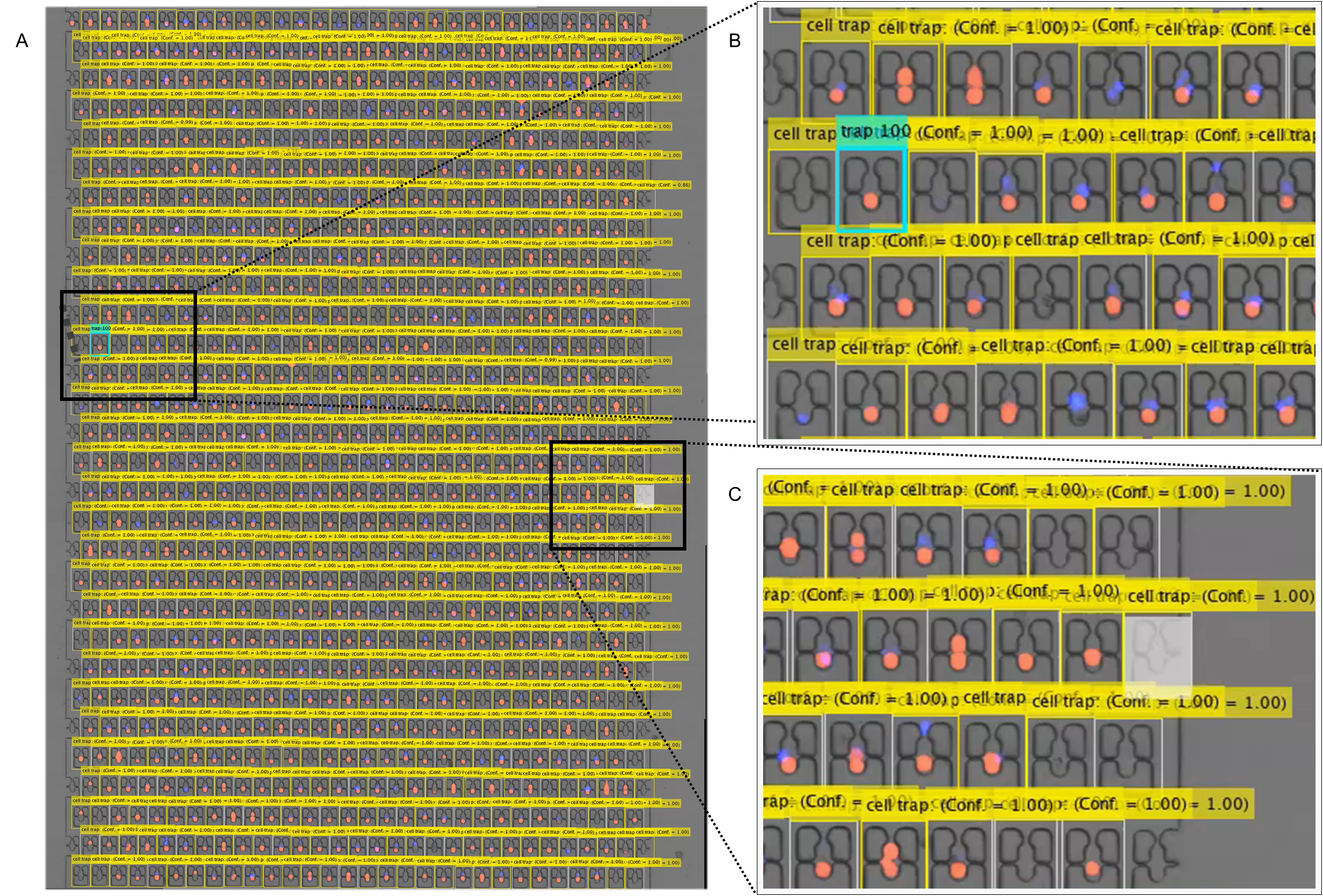


**Fig S5. R-CNN1 was used to successfully identify, track and screen cell traps related to Figure 2A (panel 1 and 2) and Figure 2B RCNN-1 “Object Detection”.**

**(A)** The stitched image of the whole microfluidic cell trap device with superimposed boxes indicating positive identification by R-CNN1 and confidence levels. The R-CNN1 object detector network was naïve to the size of the image, the number of traps in the image, any movement of the traps between frames and the dataset that was being used. Not all datasets were used to generate ground truth data to train the object detector, but the detector nevertheless demonstrated a high degree of detection. **(B)** The traps are enumerated and tracked to ensure consistency throughout the entire experiment. Trap 100 is indicated with a blue box.  **(C)** Any traps which cannot be tracked continuously across all timepoints in the 24-hour period are marked and removed from the analysis.


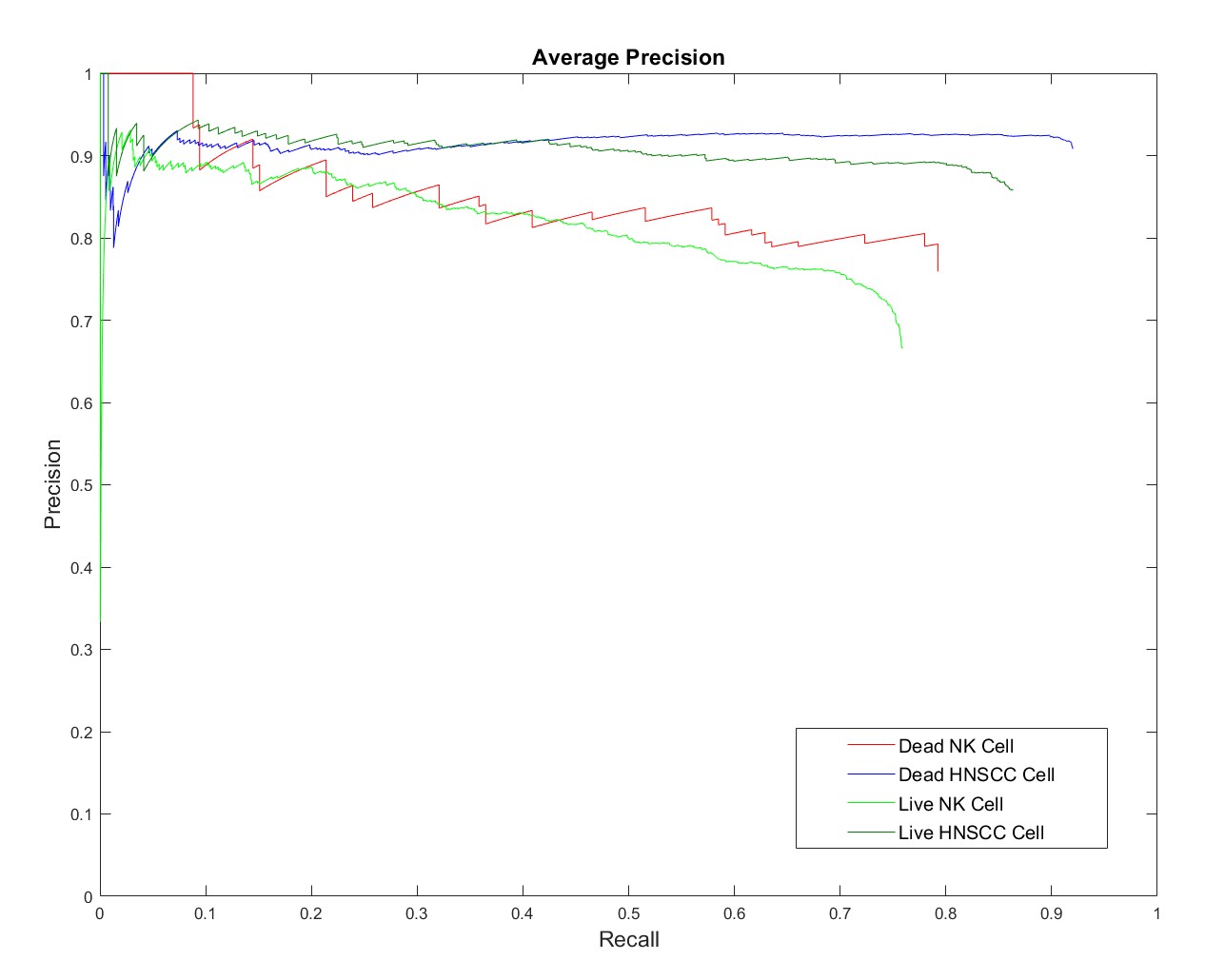


**Fig S6. Recall/Precision curves for each object class identified by R-CNN2.**

The precision (defined as the ratio of true positive instances to all positive instances of objects in the detector) for each object class was determined at various levels of recall (defined as the ratio of true positive instances to the sum of true positives and false negatives). Here, the average precision provides an estimate of the object detector’s ability to find all relevant objects (recall) and classify them correctly (precision). The most important role of R-CNN2 was identifying NK-mediated cytotoxicity of HNSCC tumor cells, and the average precision for both live and dead HNSCC cells was above 0.9 for a large range of recall values and object detection was verified visually by a human operator for a random sampling of images. The number of cells present in each cell trap was assumed constant throughout the experiment, and was verified using single channel signals from the first frame recorded in each experiment for the tumor cells and NK cells and the MATLAB function *imfindcircles* which uses a Circular Hough Transform (CHT) based algorithm to locate circular shaped objects. At each stage, a visual confirmation of a random sampling of the results was used to ensure accuracy.


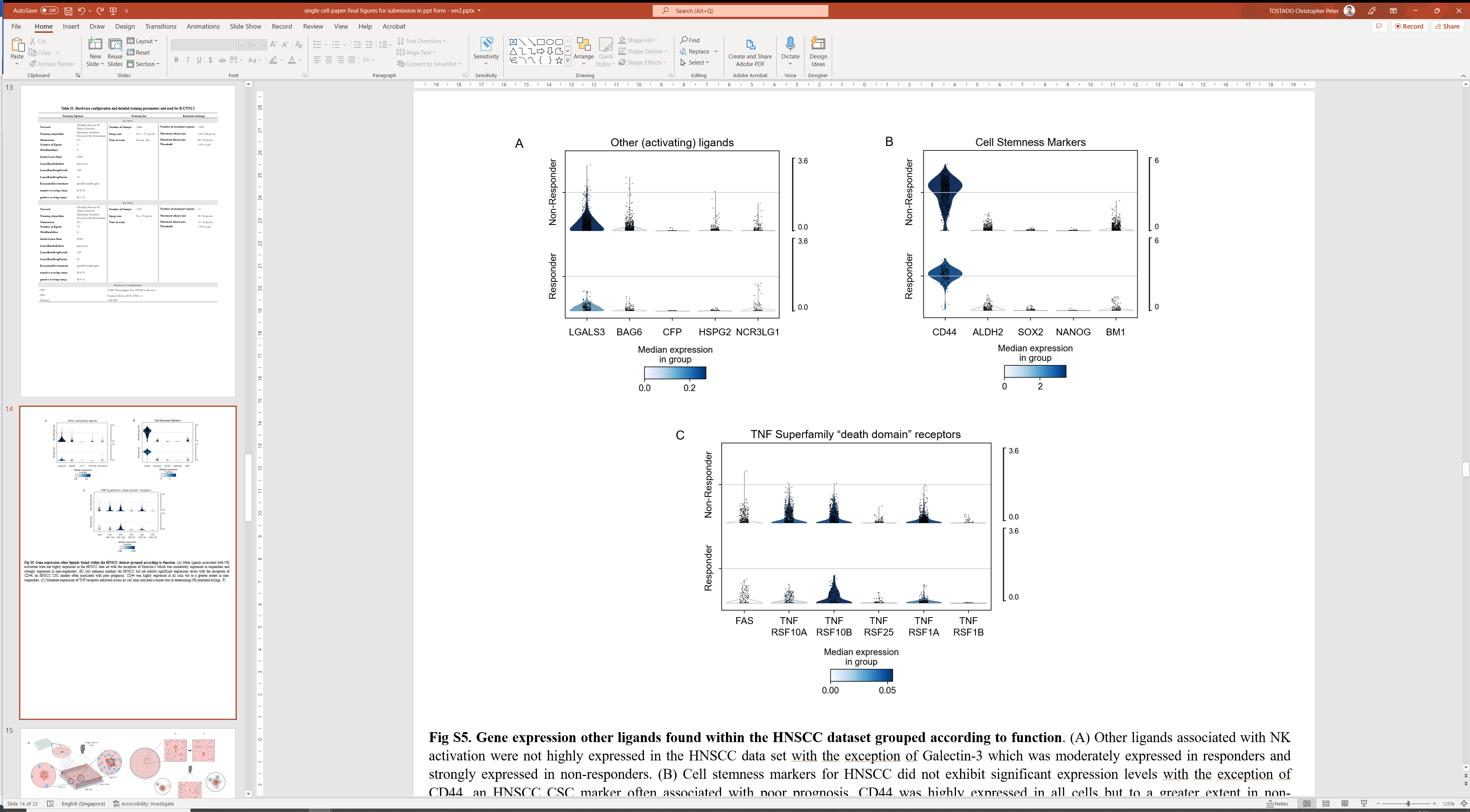


**Fig S7. Gene expression of additional ligands found within the HNSCC dataset grouped according to function related to Figure 4**.

(A) Additional ligands associated with NK activation were not highly expressed in the HNSCC data set with the exception of Galectin-3 which was moderately expressed in responders and strongly expressed in non-responders. (B) Cell stemness markers for HNSCC did not exhibit significant expression levels with the exception of CD44, an HNSCC CSC marker often associated with poor prognosis. CD44 was highly expressed in all cells but to a greater extent in non-responders. (C) Moderate expression of TNF rceptors exhibited across all cell lines indicated a minor role in determining NK-mediated killing.

Table S1. Hardware configuration and detailed training parameters used for R-CNN1/2
